# Quantifying the Evolutionary Conservation of Genes Encoding Multidrug Efflux Pumps in the ESKAPE Pathogens To Identify Antimicrobial Drug Targets

**DOI:** 10.1128/mSystems.00024-18

**Published:** 2018-04-17

**Authors:** Lauren E. Brooks, Sabah Ul-Hasan, Benjamin K. Chan, Mark J. Sistrom

**Affiliations:** a School of Natural Sciences, University of California Merced, Merced, California, USA; b Department of Ecology and Evolutionary Biology, Yale University, New Haven, Connecticut, USA; California State University, Northridge

**Keywords:** antibiotic resistance, drug resistance evolution, efflux pumps, pathogens

## Abstract

Increasing rates of antibiotic-resistant bacterial infection are one of the most pressing contemporary global health concerns. The ESKAPE pathogens (Enterococcus faecium, Staphylococcus aureus, Klebsiella pneumoniae, Acinetobacter baumannii, Pseudomonas aeruginosa, and *Enterobacter* species) have been identified as the leading global cause of multidrug-resistant bacterial infections, and overexpression of multidrug efflux (MEX) transport systems has been identified as one of the most critical mechanisms facilitating the evolution of multidrug resistance in ESKAPE pathogens. Despite efforts to develop efflux pump inhibitors to combat antibiotic resistance, the need persists to identify additional targets for future investigations. We evaluated evolutionary pressures on 110 MEX-encoding genes from all annotated ESKAPE organism genomes. We identify several MEX genes under stabilizing selection—representing targets which can facilitate broad-spectrum treatments with evolutionary constraints limiting the potential emergence of escape mutants. We also examine MEX systems being evaluated as drug targets, demonstrating that divergent selection may underlie some of the problems encountered in the development of effective treatments—specifically in relation to the NorA system in S. aureus. This study provides a comprehensive evolutionary context to efflux in the ESKAPE pathogens, which will provide critical context to the evaluation of efflux systems as antibiotic targets.

**IMPORTANCE** Increasing rates of antibiotic-resistant bacterial infection are one of the most pressing contemporary global health concerns. The ESKAPE pathogen group represents the leading cause of these infections, and upregulation of efflux pump expression is a significant mechanism of resistance in these pathogens. This has resulted in substantial interest in the development of efflux pump inhibitors to combat antibiotic-resistant infections; however, no widespread treatments have been developed to date. Our study evaluates an often-underappreciated aspect of resistance—the impact of evolutionary selection. We evaluate selection on all annotated efflux genes in all sequenced ESKAPE pathogens, providing critical context for and insight into current and future development of efflux-targeting treatments for resistant bacterial infections.

## Abstract (precis)

Increasing rates of antibiotic-resistant bacterial infection are one of the most pressing contemporary global health concerns. The ESKAPE pathogen group represents the leading cause of these infections, and upregulation of efflux pump expression is a significant mechanism of resistance in these pathogens. This has resulted in substantial interest in the development of efflux pump inhibitors to combat antibiotic-resistant infections; however, no widespread treatments have been developed to date. Our study evaluates an often-underappreciated aspect of resistance—the impact of evolutionary selection. We evaluate selection on all annotated efflux genes in all sequenced ESKAPE pathogens, providing critical context for and insight into current and future development of efflux-targeting treatments for resistant bacterial infections.

## INTRODUCTION

The widespread and inappropriate use of antibiotics as therapeutic agents for humans and domestic animals and as growth promoters in agriculture has led to an increased prevalence of multidrug-resistant (MDR) bacteria in the environment. Shortcomings in the pipeline for the discovery, development, and approval of new antibiotics to treat the array of pathogens resistant to existing antibiotics exacerbate the looming health crisis (1). The threat to public health is immediate (2), with MDR infections leading to 700,000 deaths globally in 2016 (3). If the trajectory continues, MDR cases are expected to rise to 10 million by 2050—more fatalities than both cancer and HIV/AIDS combined cause today (3).

The ESKAPE pathogens (Enterococcus faecium, Staphylococcus aureus, Klebsiella pneumoniae, Acinetobacter baumannii, Pseudomonas aeruginosa, and *Enterobacter* spp.), noted for their propensity to “escape” the inhibitory action of traditional antibiotic drugs, cause a significant proportion of nosocomial and biofilm-mediated infections (4–6). Upregulated expression of multidrug efflux (MEX) pumps is one mechanism by which the ESKAPE pathogens become resistant. These MEX pumps act by expelling compounds, including antibiotics, from the intracellular compartment/intermembrane space at a high rate, preventing drug concentrations from reaching inhibitory concentrations (7–12). Six superfamilies of MEX pumps have been identified: the ATP-binding cassette (ABC) family, the small multidrug resistance (SMR) family, the major facilitator superfamily (MFS), the resistance-nodulation-division (RND) family, the multidrug and toxic compound extrusion (MATE) family, and the recently recognized sixth superfamily, the proteobacterial antimicrobial compound efflux (PACE) family, although this group of MEX pumps is known only to efflux cationic biocides (13, 14).

As MEX pump upregulation is critical to the evolution of MDR phenotypes across a broad array of pathogens, MEX pumps are attractive targets for drug development and discovery (15). Efflux pump inhibitors (EPIs) can be combined with antimicrobial agents in order to treat otherwise MDR infections (16), rendering previously resistant bacteria susceptible to multiple classes of antibiotic agents (17). While many potential EPIs have been described in the literature (16–19), challenges associated with EPIs as therapeutic compounds, including toxicity to patients, have prevented their widespread use.

Despite these challenges, there has been a renewed interest in these compounds to treat MDR infections (15). Studies have taken steps to address substrate specificity (20, 21), but few studies have considered the conservation of genes encoding efflux pumps at the strain level (18, 22, 23). Examining how these genes are conserved prior to EPI development can ensure that potential EPIs will target the widest range of pathogenic strains and minimize the likelihood of promoting escape mutants.

In this study, we take an evolutionary approach to evaluate selection across MEX systems in the ESKAPE pathogens to identify genes described by models of purifying selection (i.e., evolution that eliminates deleterious alleles from a population), stabilizing selection (i.e., selection favoring intermediate rather than extreme variants), neutral selection (i.e., stochastic evolution), and diversifying selection (i.e., selection favoring the tails of a trait distribution resulting in adaptive divergence). In doing so, we examine the breadth of likely applications for potential EPIs targeting each of these specific MEX systems. This identification of conservation provides a comprehensive evolutionary context to all MEX pumps in the ESKAPE pathogens, along with insights into the evolutionary origins of these important structures.

## RESULTS

We took an *in silico* approach to identify genes encoding ESKAPE pathogen efflux pumps undergoing evolutionary pressure. Using only those MEX genes with previously demonstrated involvement in antibiotic resistance, chromosomal genes encoding complete or partial efflux pumps were screened for evidence of selection by calculating the ratio of nonsynonymous to synonymous mutations (*dN/dS*) and evaluating coevolution with other efflux pump genes from the same organism, using comparisons of phylogenetic topology evaluated by pairwise calculation of Robinson-Foulds (RF) distances.

**FIG 1:**
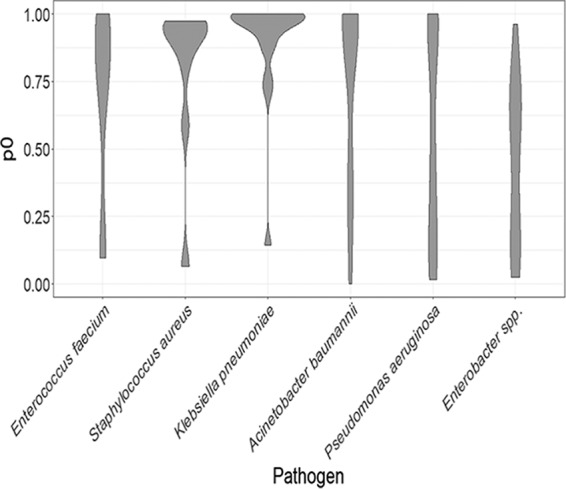
Distribution of proportion of variable sites which fit a model of stabilizing selection (*p*_0_ scores) calculated for all MEX pump-encoding genes from each pathogen. The width of each object represents the density of the data for each pathogen. This illustrates a general pattern of high conservation of MEX genes in S. aureus and K. pneumoniae, with broader variation in other groups.

Preliminary analysis of the selected genes for DNA polymorphisms revealed overall low diversity as determined by the proportion of variable sites (see Table S2 in the supplemental material). An assessment of evolutionary pressure acting upon the selected genes revealed that across genes from all ESKAPE pathogens, there was evidence for selection at a high number of sites (proportion of sites at which there is evidence for purifying selection [*p*_0_ > 90%]; see Fig. 3 and Table S4) for nearly half (51/110) of efflux pump-associated genes, although the distribution and ranges of *p*_0_ values were highly variable across each pathogen (Fig. 1; see also Fig 3 and Table S3). Ten genes were classified as being under moderate selection (75% < *p*_0_ < 90%), with the remaining 49 genes analyzed having a low (*p*_0_ < 75%) proportion of sites detected as being under selection.

Of the genes indicating low proportions of sites under purifying selection, 38 exhibited statistically significant evidence for purifying selection based on the likelihood-ratio test of a nearly neutral model (M1a), assuming only neutral and purifying selection, and a positive pressure model (M2a) (Table S4). Statistically significant purifying selection was also observed for two genes classified as under moderate selection as well as seven classified as under high selection.

To identify potential targets for EPIs, the selection models for genes of each pathogen were examined more closely. Of the selected efflux pump genes from E. faecium, *msrC* had the highest evidence for selection, with all sites classified as under selection. Moderate selection was observed for *efrA* and *efmA*, while evidence for positive selection was observed for *efrB*.

Evidence for purifying selection was found in S. aureus MDR efflux pump genes, with six of the nine genes having *p*_0_ values greater than 90% (Table S4). Not among these is the gene encoding the well-characterized MFS efflux pump NorA (24, 25), which was under moderate selection, along with two less-studied genes, *lmrS* and *sav1866*. Genes fitted better by the more complex model M2a included *norA*, *lmrS*, and *mepA*.

All identified efflux pump genes for K. pneumoniae were of the RND superfamily of efflux pumps. Purifying selection was identified for the majority (13/18) of K. pneumoniae genes, consisting of all complete RND systems except for the copper efflux system, *cusABCF*. The gene *eefC*, encoding the outer membrane protein for the *eefABC* system, was classified as having moderate selection but was close to the determined classification cutoff for high levels of purifying selection (*p*_0_ = 0.88).

A. baumannii also had a high number of genes identified as highly conserved (14/21) but not the *adeABC* efflux pump system known to not be well expressed in wild-type strains. Of the genes defined in this paper as experiencing moderate or low levels of selection, half were of the MFS superfamily of efflux pumps, while the remaining four consisted of *aceI*, a member of the PACE superfamily, and all three components of the *adeABC* RND pump. No genes demonstrated a statistically significant difference in model fit between the two models.

With 39 identified efflux pump genes, including 36 of the RND superfamily, P. aeruginosa had the highest count of genes analyzed, resulting in a wide range of *p*_0_ values (Fig. 1). These values exhibited three main clusters, corresponding to a group of high, moderate, and low selection. Included among the highly conserved genes was *oprM*, the outer membrane protein affiliated with at least four identified RND efflux pump systems in P. aeruginosa. Additionally, high *p*_0_ values were calculated for several complete efflux pump systems, such as *mexVW-oprM* and *triABC-oprM*/*triABC-opmH*, as well as genes encoding the single-protein efflux pumps PmpM and EmrE. Despite these exceptions, the majority of efflux pump genes are not strongly under selection, with 23/39 genes being classified as low or moderate. Of the genes classified as being under low selection, all but three exhibited significant evidence for positive selection (Table S4).

Unlike other ESKAPE pathogens, *Enterobacter* spp. are a grouping based on the entire genus, resulting in genomes from multiple operational taxonomic units compared in this analysis. Genes identified for this grouping had only one representative identified as under high selection (*acrD*), one under moderate selection (*eefC*), and all 16 other genes classified as under low selection, the majority of which (11/16) showed evidence for statistically significant positive selection.

**FIG 2:**
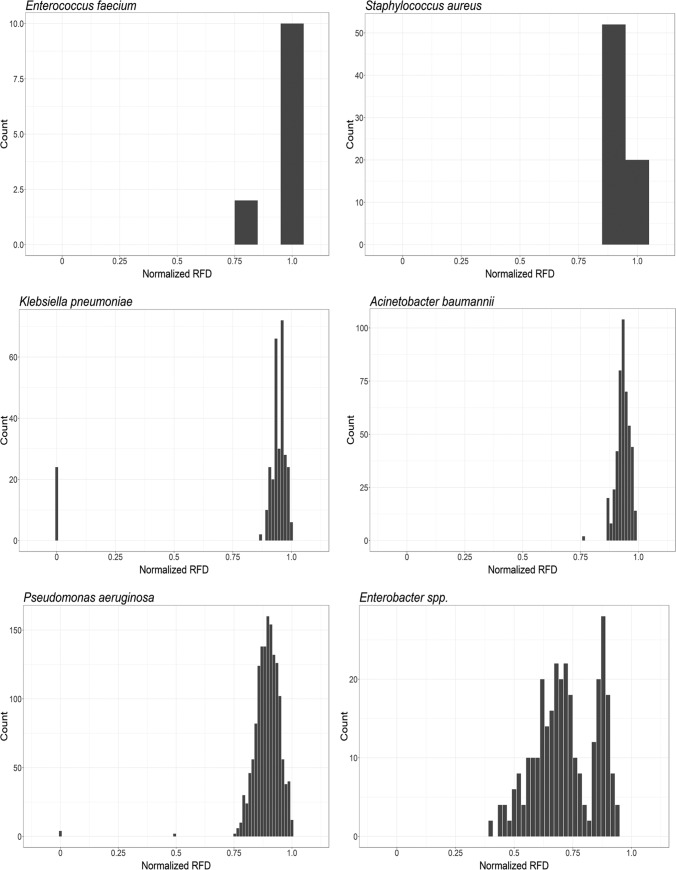
Distribution of Robinson-Foulds distances (RFD) calculated from phylogenetic trees generated for each gene in each pathogen. This demonstrates a general lack of support for coevolution of MEX genes in most cases, with the exception of some analogous genes in P. aeruginosa and K. pneumoniae, suggesting that these pumps may result from recent gene duplication events.

We used RF topological distances to compare gene trees for each pathogen and examine the presence of a possible shared evolutionary history. With a few notable exceptions, normalized RF scores indicate that most of the distances calculated between gene trees were high (approaching 1), indicative of independent evolutionary histories (Fig. 2). However, low levels of polymorphism were detected in many genes (Table S2), suggesting that the observed RF distances are likely impacted by a lack of phylogenetic resolution in many comparisons, potentially resulting in a limited power of the analysis in these cases (26). Thus, the observed high RF scores should be interpreted with caution.

Within the genes identified for K. pneumoniae, 24 out of 306 gene pairs had a calculated RF distance of 0, meaning phylogenetic topologies identical and suggesting possible coevolution (Fig. 2). These genes having RF distances of zero included sets of the same RND components: periplasmic adaptors *acrA*, *eefA*, and *acrE*; inner membrane transporters *acrB*, *acrD*, *acrF*, and *eefB*; and outer membrane protein genes *cusC* and *eefC*. Additionally, phylogenetic topologies were identical for the components of the *mdtABC* and *oqxA*. Identical topologies were also identified between two gene tree comparisons, *mexX-mexG* and *mexY-mexH*, from P. aeruginosa. All other pathogens had ranges of RF distances that did not include zero (Fig. 2).

## DISCUSSION

Despite being a fundamentally evolutionary process, the role of evolution is often underappreciated in the development of strategies to combat antibiotic resistance. Examining potential drug targets from an evolutionary perspective provides insight into the likely effectiveness of treatments impacting a selected target. Given the pressing need and high costs for establishing new ways to treat antibiotic-resistant infections, additional screening of potential treatments can prevent investing of resources in drugs that are unlikely to be broadly effective at treating resistant infections. Consideration of the results from this analysis provides a basis for the development of evolutionary robust and broadly effective methods to treat MDR infections in the face of a looming health crisis.

EPIs targeting the efflux pumps encoded by genes that we have identified as being under purifying selection have already been developed for several MEX systems. For example, *msrC*, the only gene identified from E. faecium as a potential good target, has previously been proposed as a potential drug target, as reductions in the MICs of macrolide antibiotics were demonstrated (27). However, questions regarding the distribution of this gene raised questions regarding the potential of *msrC* as a drug target (28). This analysis suggests that not only is some form of this gene present in all sequenced genomes of E. faecium but also that the gene is under stabilizing selection in these genomes and could make a good target for treating MDR E. faecium.

**FIG 3:**
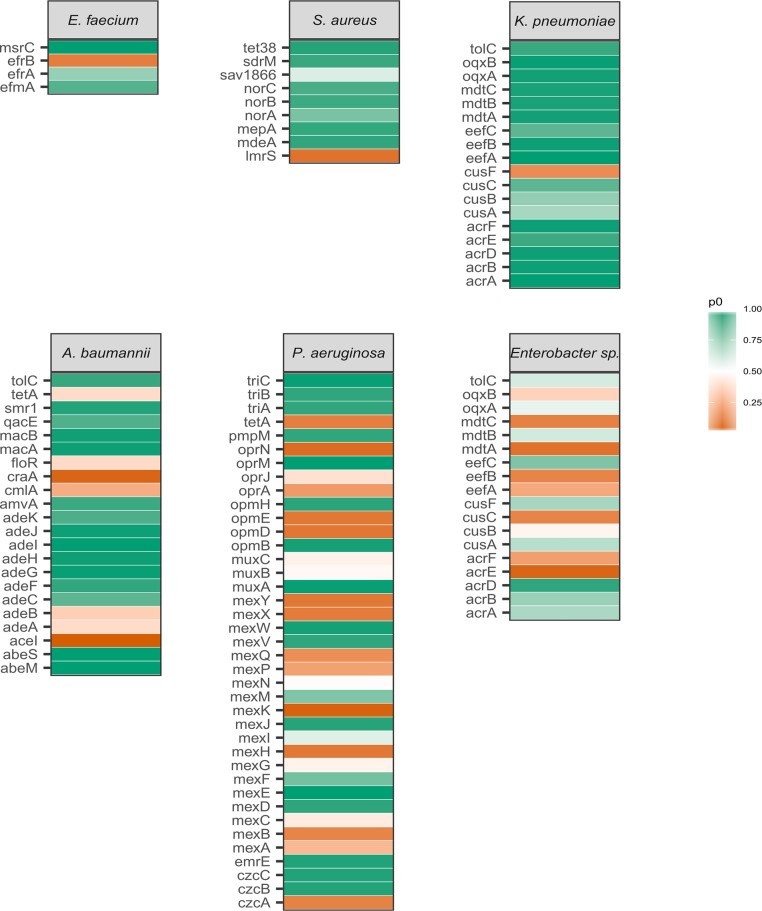
A selection map showing the ESKAPE species, genes tested, and proportion of sites under stabilizing selection. Color indicates the relative strength of stabilizing selection on each gene in each species.

Although *Enterobacter* spp. and K. pneumoniae share the same list of efflux pumps (Fig. 3), no genes were identified as being under selection for *Enterobacter* spp. while most genes from K. pneumoniae were classified as highly conserved. It is likely that the genus-level analysis of *Enterobacter* spp. resulted in higher genetic variation (average nucleotide diversity = 0.16) than that for K. pneumoniae (average nucleotide diversity = 0.02). This discrepancy limits the ability to directly compare these groups, further raising questions regarding the grouping of this genus as one class of ESKAPE pathogens, as individual species may have different mechanisms and evolutionary pressures acting upon them.

The *acrAB-tolC* system in Escherichia coli has received a lot of attention as a potential drug target to treat resistant infections (17, 29), including the use of the FDA-approved antipsychotic medication pimozide (30). This system is also found in both *Enterobacter* spp. and K. pneumoniae, is comparable with the *adeABC* and *adeIJK* systems in A. baumannii (31), and was found in this analysis to likely to be a good candidate for treatment with EPIs. While a number of additional genes were identified here as good candidates from K. pneumoniae and A. baumannii, little research has gone into developing EPIs for additional systems.

EPIs have been developed for treating MDR S. aureus, but none have proven to be a reliable treatment. One common target for EPI development is *norA*, shown in this analysis to be highly variable and under positive selection (see Table S4 in the supplemental material). Large amounts of variation in this gene have been observed previously (32, 33), possibly contributing to the failures of tested EPIs. Consideration of evolutionary selection has the potential to greatly increase the success of future EPI trials. Thus, while *norA* is highly effective at the removal of antibiotics, it may be difficult to target with EPIs due to genetic inconsistency.

MEX systems in P. aeruginosa have been heavily studied, as they are prevalent in MDR strains. However, our findings suggest a lack of evolutionary pressure observed for a majority of these systems. One possible explanation for the discrepancy between what is known to be an important set of systems and the lack of evolutionary pressure is the possibility of redundancy within the efflux pumps. The sheer volume of efflux pump systems, many of which have components that are interchangeable with other systems, might alleviate the evolutionary pressure observed for many of the other systems, making MEX systems from P. aeruginosa more variable across strains.

In addition to screening for selective pressure, we used RF distances to measure the similarity/dissimilarity of phylogenies for each gene, providing for evidence of shared or independent evolutionary history. As MEX pumps are multiprotein/gene structures, coevolution indicates a tight link between pump components, and thus, mutations in individual components of MEX pumps affect the function of the structure as a whole. Conversely, independent evolutionary histories may indicate that pumps had been recently coopted from other functions and are able to respond rapidly to novel evolutionary selection.

Despite limitations due to low overall genetic diversity in the genes analyzed (see Table S2), a number of RF comparisons in K. pneumoniae and P. aeruginosa demonstrated identical phylogenetic topologies. These genes are largely analogous genes in distinct MEX pump systems, which is indicative of these distinct pump systems arising from relatively recent gene duplication events. The possibility of spontaneous emergence of novel MEX systems may pose challenges for treating and monitoring MDR organisms, but the shared evolutionary history of distinct MEX systems may also mean that similar strategies may be used to target multiple MEX pumps in these pathogens.

## MATERIALS AND METHODS

### Data collection.

Publicly available genome coding sequences for each ESKAPE pathogen were downloaded from NCBI (accessed on 14 July 2017). We selected complete genomes only, resulting in 545 genomes from E. faecium (*n* = 26), S. aureus (*n* = 168), K. pneumoniae (*n* = 137), A. baumannii (*n* = 79), P. aeruginosa (*n* = 80), and *Enterobacter* spp. (*n* = 55), including representatives from available operational taxonomic units within the genus (see Table S1 in the supplemental material). Downloaded coding sequences were converted to Basic Local Alignment Search Tool databases using BLAST+ (34).

10.1128/mSystems.00024-18.1 TABLE S1  List of GenBank accession numbers for genomes used in this study. Download TABLE S1, XLSX file, 0.1 MB. Copyright © 2018 Brooks et al. 2018 Brooks et al. This content is distributed under the terms of the Creative Commons Attribution 4.0 International license.

10.1128/mSystems.00024-18.2 TABLE S2  Polymorphism results for each gene in each species. Download TABLE S2, PDF file, 0.1 MB. Copyright © 2018 Brooks et al. 2018 Brooks et al. This content is distributed under the terms of the Creative Commons Attribution 4.0 International license.

10.1128/mSystems.00024-18.3 TABLE S3  Normalized Robinson-Foulds scores for pairwise comparisons between phylogenies for each gene in each species. Download TABLE S3, PDF file, 0.5 MB. Copyright © 2018 Brooks et al. 2018 Brooks et al. This content is distributed under the terms of the Creative Commons Attribution 4.0 International license.

10.1128/mSystems.00024-18.4 TABLE S4  Results from selection analyses; Stat, both *p* (proportion of sites which match each model of selection) and ω (*dN/dS* ratio for the variable sites which fit into each model of selection) for purifying, neutral, and positive; *P* value, probability value for likelihood-ratio test statistic. Download TABLE S4, PDF file, 0.3 MB. Copyright © 2018 Brooks et al. 2018 Brooks et al. This content is distributed under the terms of the Creative Commons Attribution 4.0 International license.

Chromosomally located genes (*n* = 110) encoding efflux pumps and their components from all of the ESKAPE pathogens, as listed in the work of Li et al. (35), were included in the analysis. The amino acid sequence for each of the identified genes was downloaded from the UniProt knowledge base (36) using the reference genome for each pathogen. These sequences were then used to identify analogous genes from the downloaded coding sequences for each pathogen using BLAST+ (34). Duplicate gene sequences within a genome were screened for similarity and in all cases were identical, allowing only one identified gene sequence to be used for analysis.

### Data analysis.

Sequences within the genomes with the top hit identified from BLAST+ output from each genome were aligned for each gene using MUSCLE v3.8.31 (37). The resulting alignments were used to analyze individual genes for DNA polymorphisms with DnaSP v5 (38) and to construct unrooted maximum likelihood trees using RAxML (39) with a bootstrap value of 1,000.

Genes were screened for evidence of evolutionary pressures using the Phylogenetic Analysis by Maximum Likelihood (40) software package. Positive selection was tested for each gene based on the ratio of nonsynonymous to synonymous mutations per site (ω = *dN/dS*) using the codeml program. Variable sites in each gene alignment were fitted to both a nearly neutral model (M1a), assuming only neutral and purifying selection, and a positive-pressure model (M2a), allowing sites to be classified as either purifying, neutral, or under positive selection. All models were fitted using a single ω value for all lineages and average nucleotide frequencies at the third codon position.

Genes were assessed for evidence of evolutionary pressure by determining the proportion of sites at which there is evidence for purifying selection (*p*_0_) under M2a. Sites with high *p*_0_ values (*p*_0_ > 90%) are classified here as highly conserved and thus are more likely to be stable drug targets across multiple pathogen strains. The χ^2^ analysis of likelihood ratios of the two models was used to calculate the statistical significance of fitting the more complex model (M2a) compared to the simpler model (M1a) to suggest an observed positive, or diversifying, for the genes.

To assess if any combination of genes exhibited coevolution, Robinson-Foulds (RF) distances (41) were calculated pairwise for all phylogenies within ESKAPE groups using the phytools package (42) in R statistical software (43).
