# Supplementary material for: Quantifying the Evolutionary Conservation of Genes Encoding Multidrug Efflux Pumps in the ESKAPE Pathogens To Identify Antimicrobial Drug Targets
Source: mSystems. 2018 Apr 17;3(3):e00024-18. doi: 10.1128/mSystems.00024-18 (PMC5904435; doi:10.1128/mSystems.00024-18)
Supplement: TABLE S2 [file sys003182224st2.pdf]

Table S2: Polymorphism results for each gene in each species. n- number of individuals sampled, Sites – number of base pairs in each gene, MissingSites – number of sites with alignment gaps, S – number of segregating sites, InvarSites – number of invariant sites, Eta – total number of mutations, Hap – number of haplotypes Hd – haplotype diversity, VarHd – variance in haplotype diversity, Pi – Nucleotide diversity, AvNumDif – average pair wise nucleotide difference per sequence, ThetaNuc –estimated population mutation rate per site ThetaG – mutation parameter per sequence Zns - ZnS statistic, G+Cn - GC content at noncoding positions, G+Gc – GC content at coding positions, G+Ctot – GC content, TajimaD – Tajima's D statistic, SigD – significance of Tajima's D, FuLiD* - Fu and Li's D* statistic, SigD* - significance of Fu and Li's D*, FuLiF* - Fu and Li's F* statistic, SigF – significance of Fu and Li's F* FuFs – Fu's Fs statistic

Gene	Organism	Region	Ingroup	Outgroup	n	Sites	NetSites	MissingSit	S	InvarSites	Prop_Var	Eta	Hap	Hd	VarHd	Pi	VarPi	AvNumDif	ThetaNuc	ThetaG	ZnS	G+Cn	G+Gc	G+Ctot	TajimaD	SigD	FuLiD*	SigD	FuLiF*	SigF	FuFs
tolC	Acinetobac	1-1347	79	0	79	1347	1332	15	71	1261	0.053303	73	16	0.677	0.0033	0.00953	n.d.	12.6946	0.011093	14.7764	0.1144	0.4082	0	0.4082	-0.4691	n.s.	-0.7506	n.s.	-0.7647	n.s.	5.958
macB	Acinetobac	1-1995	79	0	79	1995	1995	0	222	1773	0.111278	237	16	0.665	0.00348	0.012871	n.d.	25.6774	0.024046	47.9726	0.3029	0.4475	0	0.4475	-1.5993	#	-4.5744	**	-4.0028	**	17.387
macA	Acinetobac	1-1344	79	0	79	1344	867	477	61	806	0.070358	63	16	0.661	0.00337	0.011891	n.d.	10.3096	0.014708	12.7522	0.1588	0.3993	0	0.3993	-0.6331	n.s.	-1.0306	n.s.	-1.0457	n.s.	3.91
adeB	Acinetobac	1-3294	79	0	79	3294	1093	2201	612	481	0.559927	762	19	0.665	0.00349	0.057182	n.d.	62.4995	0.141117	154.241	0.4882	0.4139	0	0.4139	-2.0685	*	2.2337	**	0.5001	n.s.	36.31
adeJ	Acinetobac	1-3177	79	0	79	3177	3177	0	83	3094	0.026125	86	19	0.68	0.00337	0.003032	n.d.	9.6323	0.005479	17.4078	0.0702	0.424	0	0.424	-1.4977	n.s.	-1.7256	n.s.	-1.9593	#	1.547
adeG	Acinetobac	1-3180	79	0	79	3180	3180	0	277	2903	0.087107	300	17	0.676	0.00326	0.019678	n.d.	62.5771	0.019096	60.7248	0.137	0.4467	0	0.4467	0.1053	n.s.	0.7067	n.s.	0.5463	n.s.	40.899
abeM	Acinetobac	1-1347	79	0	79	1347	837	510	32	805	0.038232	32	17	0.677	0.00328	0.009622	n.d.	8.0532	0.007739	6.4773	0.1711	0.4032	0	0.4032	0.7661	n.s.	1.0518	n.s.	1.1276	n.s.	1.507
craA	Acinetobac	1-1440	79	0	79	1440	1038	402	418	620	0.402697	426	17	0.677	0.0033	0.017739	n.d.	18.4135	0.083072	86.2292	0.8478	0.373	0	0.373	-2.7247	***	-8.7912	**	-7.4928	**	9.836
cmlA	Acinetobac	1-1470	79	0	79	1470	1008	462	728	280	0.722222	1000	20	0.681	0.0034	0.114718	n.d.	115.6355	0.20081	202.416	0.3916	0.4462	0	0.4462	-1.4927	n.s.	-1.2201	n.s.	-1.6031	n.s.	57.016
floR	Acinetobac	1-1372	79	0	79	1372	1055	317	744	311	0.705213	1024	19	0.681	0.0034	0.119361	n.d.	125.926	0.196468	207.274	0.4338	0.4449	0	0.4449	-1.3666	n.s.	2.4317	**	0.9902	n.s.	63.374
tetA	Acinetobac	1-1555	79	0	79	1555	751	804	599	152	0.797603	952	17	0.776	0.00168	0.345732	n.d.	259.6449	0.256591	192.7	0.3296	0.4448	0	0.4448	1.2093	n.s.	2.4726	**	2.327	**	n.d.
amvA	Acinetobac	1-1512	79	0	79	1512	1218	294	163	1055	0.133826	175	19	0.692	0.00319	0.022754	n.d.	27.7147	0.029083	35.4228	0.2046	0.4069	0	0.4069	-0.7448	n.s.	1.0026	n.s.	0.3351	n.s.	14.526
adeC	Acinetobac	1-1468	79	0	79	1468	1322	146	453	869	0.342663	512	18	0.665	0.00347	0.130489	n.d.	172.5063	0.078394	103.637	0.6799	0.3873	0	0.3873	2.3056	*	1.7783	**	2.4013	**	n.d.
adeK	Acinetobac	1-1468	79	0	79	1468	1400	68	443	957	0.316429	460	18	0.692	0.00319	0.014554	n.d.	20.3759	0.066508	93.1114	0.8627	0.4231	0	0.4231	-2.7081	***	-8.6659	**	-7.3948	**	10.258
adeH	Acinetobac	1-1452	79	0	79	1452	1388	64	104	1284	0.074928	105	18	0.665	0.00347	0.009466	n.d.	13.1392	0.015312	21.2537	0.1407	0.4444	0	0.4444	-1.2894	n.s.	-2.9653	*	-2.7383	*	4.618
aceI	Acinetobac	1-577	79	0	79	577	358	219	162	196	0.452514	168	10	0.591	0.00352	0.026273	n.d.	9.4057	0.094989	34.0059	0.8803	0.3504	0	0.3504	-2.474	**	2.1316	**	0.2724	n.s.	9.074
adeA	Acinetobac	1-1323	79	0	79	1323	3	1320	1	2	0.333333	1	2	0.184	0.00294	0.061452	n.d.	0.1844	0.067472	0.2024	n.a.	0.2996	0	0.2996	-0.1075	n.s.	0.5078	n.s.	0.3781	n.s.	0.353
adeF	Acinetobac	1-1992	79	0	79	1992	1977	15	175	1802	0.088518	179	19	0.773	0.00209	0.010179	n.d.	20.124	0.018327	36.2325	0.196	0.4465	0	0.4465	-1.5224	n.s.	-3.187	*	-2.9977	*	8.936
adeI	Acinetobac	1-1251	79	0	79	1251	1248	3	17	1231	0.013622	17	13	0.669	0.00307	0.002471	n.d.	3.0841	0.002757	3.4411	0.1404	0.4287	0	0.4287	-0.3025	n.s.	-2.1324	#	-1.7528	n.s.	-1.181
qacE	Acinetobac	1-348	79	0	79	348	330	18	158	172	0.478788	180	14	0.709	0.0027	0.229768	n.d.	75.8234	0.110409	36.4349	0.8164	0.4209	0	0.4209	3.7024	***	2.3565	**	3.4976	**	56.129
smr1	Acinetobac	1-697	79	0	79	697	197	500	23	174	0.116751	23	11	0.641	0.00333	0.014785	n.d.	2.9127	0.023632	4.6556	0.2392	0.4163	0	0.4163	-1.1377	n.s.	-0.1081	n.s.	-0.5846	n.s.	-0.228
abeS	Acinetobac	1-330	79	0	79	330	330	0	46	284	0.139394	50	14	0.673	0.00317	0.020102	n.d.	6.6336	0.030669	10.1208	0.4092	0.3527	0	0.3527	-1.1237	n.s.	1.2875	n.s.	0.4072	n.s.	2.068
cusF	Enterobact	1-409	53	0	53	409	238	171	170	68	0.714286	309	26	0.876	0.00137	0.208566	n.d.	49.6386	0.286097	68.091	0.1691	0.4769	0	0.4769	-0.977	n.s.	0.3706	n.s.	-0.1774	n.s.	7.58
mdtC	Enterobact	1-3397	53	0	53	3397	2791	606	1979	812	0.709065	3051	42	0.982	0.0001	0.125405	n.d.	350.0065	0.240887	672.3161	0.2945	0.6129	0	0.6129	-1.7447	#	-3.0199	*	-3.0254	*	8.882
eefB	Enterobact	1-3178	53	0	53	3178	2051	1127	1136	915	0.553876	1800	40	0.978	0.00012	0.190179	n.d.	390.0581	0.193392	396.6467	0.2391	0.6003	0	0.6003	-0.0604	n.s.	-0.0156	n.s.	-0.0395	n.s.	13.288
acrF	Enterobact	1-3152	53	0	53	3152	3103	49	1425	1678	0.459233	2345	41	0.981	0.0001	0.137895	n.d.	427.8882	0.16653	516.7425	0.1333	0.5448	0	0.5448	-0.6256	n.s.	0.3891	n.s.	-0.0061	n.s.	12.444
oqxB	Enterobact	1-3447	53	0	53	3447	2452	995	1697	755	0.692088	2556	41	0.981	0.0001	0.127416	n.d.	312.4253	0.229706	563.2383	0.2744	0.5948	0	0.5948	-1.6203	#	-1.3204	n.s.	-1.722	n.s.	9.374
cusB	Enterobact	1-1533	53	0	53	1533	1091	442	784	307	0.718607	1361	33	0.951	0.00036	0.217141	n.d.	236.9006	0.274894	299.909	0.2943	0.5164	0	0.5164	-0.7636	n.s.	0.4122	n.s.	-0.0528	n.s.	19.917
mdtB	Enterobact	1-3390	53	0	53	3390	2847	543	1741	1106	0.611521	2499	42	0.982	0.0001	0.113024	n.d.	321.7787	0.193424	550.6778	0.3602	0.6116	0	0.6116	-1.5124	n.s.	-2.6097	*	-2.6167	*	8.163
acrB	Enterobact	1-3151	53	0	53	3151	3110	41	1276	1834	0.410289	1860	42	0.985	0.00007	0.08537	n.d.	265.4993	0.13179	409.8682	0.1856	0.5591	0	0.5591	-1.281	n.s.	-1.4934	n.s.	-1.6909	n.s.	6.605
acrD	Enterobact	1-3156	53	0	53	3156	3105	51	1541	1564	0.496296	2418	42	0.982	0.0001	0.133903	n.d.	415.77	0.171603	532.8287	0.2062	0.5818	0	0.5818	-0.7993	n.s.	0.6557	n.s.	0.1084	n.s.	10.417
cusC	Enterobact	1-1641	53	0	53	1641	1094	547	858	236	0.784278	1506	34	0.957	0.00029	0.214798	n.d.	234.9891	0.303346	331.861	0.225	0.5277	0	0.5277	-1.0612	n.s.	0.6971	n.s.	0.0175	n.s.	17.856
tolC	Enterobact	1-1682	53	0	53	1682	1200	482	737	463	0.614167	1000	42	0.982	0.0001	0.108477	n.d.	130.172	0.183633	220.3593	0.3668	0.5535	0	0.5535	-1.4862	n.s.	-2.799	*	-2.7441	*	2.019
eefC	Enterobact	1-1458	53	0	53	1458	1178	280	945	233	0.802207	1664	41	0.981	0.0001	0.272989	n.d.	321.5813	0.311271	366.6778	0.2495	0.6079	0	0.6079	-0.4472	n.s.	1.054	n.s.	0.5647	n.s.	9.643
acrE	Enterobact	1-1199	53	0	53	1199	1131	68	643	488	0.568523	1084	40	0.977	0.00014	0.170355	n.d.	192.6713	0.211202	238.8694	0.1496	0.5587	0	0.5587	-0.7025	n.s.	0.5336	n.s.	0.065	n.s.	6.411
mdtA	Enterobact	1-1460	53	0	53	1460	1015	445	651	364	0.641379	947	42	0.982	0.0001	0.124503	n.d.	126.3701	0.205596	208.6802	0.3303	0.6061	0	0.6061	-1.4321	n.s.	-2.2616	#	-2.3247	#	1.867
eefA	Enterobact	1-1218	53	0	53	1218	980	238	642	338	0.655102	1070	39	0.976	0.00014	0.231422	n.d.	226.7939	0.240596	235.7844	0.2429	0.6076	0	0.6076	-0.1385	n.s.	1.0944	n.s.	0.7385	n.s.	9.183
oqxA	Enterobact	1-1262	53	0	53	1262	1005	257	660	345	0.656716	971	40	0.98	0.0001	0.143251	n.d.	143.9673	0.212904	213.9688	0.3357	0.5904	0	0.5904	-1.1879	n.s.	0.2644	n.s.	-0.3578	n.s.	4.175
cusA	Enterobact	1-3309	53	0	53	3309	3087	222	1678	1409	0.54357	2860	39	0.979	0.0001	0.230866	n.d.	712.6843	0.204155	630.2275	0.275	0.5666	0	0.5666	0.4761	n.s.	0.5484	n.s.	0.6236	n.s.	22.78
acrA	Enterobact	1-1250	53	0	53	1250	1135	115	566	569	0.498678	877	39	0.972	0.0002	0.115233	n.d.	130.7896	0.170269	193.2551	0.1899	0.5468	0	0.5468	-1.1732	n.s.	-1.6676	n.s.	-1.7686	n.s.	4.376
efrB	Enterococ	1-1882	23	0	23	1882	1643	239	711	932	0.432745	740	5	0.391	0.01565	0.040223	n.d.	66.087	0.122032	200.4978	0.8718	0.4024	0	0.4024	-2.7346	***	-4.5648	**	-4.6827	**	32.835
efrA	Enterococ	1-1822	23	0	23	1822	1625	197	705	920	0.433846	734	6	0.458	0.01584	0.040506	n.d.	65.8221	0.122383	198.8722	0.9011	0.3905	0	0.3905	-2.729	***	-4.5268	**	-4.65	**	29.001
msrC	Enterococ	1-1479	23	0	23	1479	1479	0	82	1397	0.055443	82	6	0.632	0.00801	0.005195	n.d.	7.6838	0.015022	22.2173	0.8265	0.3886	0	0.3886	-2.6029	***	-4.258	**	-4.3873	**	5.471
efmA	Enterococ	1-1338	23	0	23	1338	1278	60	351	927	0.274648	352	6	0.514	0.01437	0.024121	n.d.	30.8261	0.074626	95.3719	0.9857	0.4097	0	0.4097	-2.7511	***	-4.6968	**	-4.795	**	18.575
cusF	Klebsiella p	1-2983	79	0	79	2983	136	2847	120	16	0.882353	216	15	0.781	0.00082	0.310454	n.d.	42.2217	0.321484	43.7219	0.1281	0.356	0	0.356	-0.1179	n.s.	-0.537	n.s.	-0.4353	n.s.	32.361
cusB	Klebsiella p	1-2008	79	0	79	2008	905	1103	743	162	0.820994	1102	24	0.71	0.00315	0.239093	n.d.	216.3794	0.246478	223.0624	0.2338	0.4088	0	0.4088	-0.1043	n.s.	-1.46	n.s.	-1.064	n.s.	n.d.
mdtB	Klebsiella p	1-3099	79	0	79	3099	3006	93	152	2854	0.050566	157	20	0.755	0.00245	0.008723	n.d.	26.221	0.010572	31.7793	0.0851	0.3968	0	0.3968	-0.5973	n.s.	-0.9557	n.s.	-0.9695	n.s.	12.151
mdtC	Klebsiella p	1-3099	79	0	79	3099	3006	93	152	2854	0.050566	157	20	0.755	0.00245	0.008723	n.d.	26.221	0.010572	31.7793	0.0851	0.3968	0	0.3968	-0.5973	n.s.	-0.9557	n.s.	-0.9695	n.s.	12.151
acrB	Klebsiella p	1-3177	79	0	79	3177	3177	0	83	3094	0.026125	86	19	0.68	0.00337	0.003032	n.d.	9.6323	0.005479	17.4078	0.0702	0.424	0	0.424	-1.4977	n.s.	-1.7256	n.s.	-1.9593	#	1.547
acrD	Klebsiella p	1-3177	79	0	79	3177	3177	0	83	3094	0.026125	86	19	0.68	0.00337	0.003032	n.d.	9.6323	0.005479	17.4078	0.0702	0.424	0	0.424	-1.4977	n.s.	-1.7256	n.s.	-1.9593	#	1.547
acrF	Klebsiella p	1-3177	79	0	79	3177	3177	0	83	3094	0.026125	86	19	0.68	0.00337	0.003032	n.d.	9.6323	0.005479	17.4078	0.0702	0.424	0	0.424	-1.4977	n.s.	-1.7256	n.s.	-1.9593	#	1.547
eefB	Klebsiella p	1-3177	79	0	79	3177	3177	0	83	3094	0.026125	86	19	0.68	0.00337	0.003032	n.d.	9.6323	0.005479	17.4078	0.0702	0.424	0	0.424	-1.4977	n.s.	-1.7256	n.s.	-1.9593	#	1.547
oqxB	Klebsiella p	1-3180	79	0	79	3180	3180	0	277	2903	0.087107	300	17	0.676	0.00326	0.019678	n.d.	62.5771	0.019096	60.7248	0.137	0.4467	0	0.4467	0.1053	n.s.	0.7067	n.s.	0.5463	n.s.	40.899
cusC	Klebsiella p	1-1468	79	0	79	1468	1322	146	453	869	0.342663	512	18	0.665	0.00347	0.130489	n.d.	172.5063	0.078394	103.637	0.6799	0.3873	0	0.3873	2.3056	*	1.7783	**	2.4013	**	n.d.
eefC	Klebsiella p	1-1468	79	0	79	1468	1322	146	453	869	0.342663	512	18	0.665	0.00347	0.130489	n.d.	172.5063	0.078394	103.637	0.6799	0.3873	0	0.3873	2.3056	*	1.7783	**	2.4013	**	n.d.
tolC	Klebsiella p	1-1347	79	0	79	1347	1332	15	71	1261	0.053303	73	16	0.677	0.0033	0.00953	n.d.	12.6946	0.011093	14.7764	0.1144	0.4082	0	0.4082	-0.4691	n.s.	-0.7506	n.s.	-0.7647	n.s.	5.958
cusA	Klebsiella p	1-3304	79	0	79	3304	1886	1418	1189	697	0.630435	1666	22	0.696	0.00328	0.215103	n.d.	405.6842	0.178804	337.225	0.4284	0.432	0	0.432	0.7078	n.s.	0.8152	n.s.	0.924	n.s.	n.d.
acrE	Klebsiella p	1-1251	79	0	79	1251	1248	3	17	1231	0.013622	17	13	0.669	0.00307	0.002471	n.d.	3.0841	0.002757	3.4411	0.1404	0.4287	0	0.4287	-0.3025	n.s.	-2.1324	#	-1.7528	n.s.	-1.181
mdtA	Klebsiella p	1-1221	79	0	79	1221	1218	3	65	1153	0.053366	70	15	0.66	0.00334	0.01119	n.d.	13.63	0.011633	14.1691	0.1316	0.4479	0	0.4479	-0.1264	n.s.	1.1369	n.s.	0.7566	n.s.	7.82
oqxA	Klebsiella p	1-1221	79	0	79	1221	1218	3	65	1153	0.053366	70	15	0.66	0.00334	0.01119	n.d.	13.63	0.011633	14.1691	0.1316	0.4479	0	0.4479	-0.1264	n.s.	1.1369	n.s.	0.7566	n.s.	7.82
acrA	Klebsiella p	1-1251	79	0	79	1251	1248	3	17	1231	0.013622	17	13	0.669	0.00307	0.002471	n.d.	3.0841	0.002757	3.4411	0.1404	0.4287	0	0.4287	-0.3025	n.s.	-2.1324	#	-1.7528	#	-1.181
eefA	Klebsiella p	1-1251	79	0	79	1251	1248	3	17	1231	0.013622	17	13	0.669	0.00307	0.002471	n.d.	3.0841	0.002757	3.4411	0.1404	0.4287	0	0.4287	-0.3025	n.s.	-2.1324	#	-1.7528	#	-1.181
mexK	Pseudomo	1-3188	80	0	80	3188	2962	226	1055	1907	0.356178	1105	47	0.979	0.00004	0.014912	n.d.	44.1699	0.07532	223.098	0.8002	0.6653	0	0.6653	-2.7899	***	-8.8988	**	-7.5711	**	-0.197
mexY	Pseudomo	1-3201	80	0	80	3201	3034	167	1084	1950	0.357284	1155	52	0.98	0.00005	0.028957	n.d.	87.8554	0.07686	233.193	0.726	0.683	0	0.683	-2.1684	**	0.9793	n.s.	-0.4281	n.s.	2.52
czcA	Pseudomo	1-3197	80	0	80	3197	3097	100	1076	2021	0.347433	1131	49	0.98	0.00004	0.012447	n.d.	38.5497	0.073732	228.3474	0.8073	0.6719	0	0.6719	-2.8916	***	-8.8704	**	-7.603	**	-1.988
mexB	Pseudomo	1-3273	80	0	80	3273	2767	506	1336	1431	0.482833	1546	42	0.97	0.00006	0.020418	n.d.	56.4968	0.112806	312.1354	0.496	0.6488	0	0.6488	-2.8517	***	-6.6153	**	-6.022	**	4.016
mexQ	Pseudomo	1-3232	80	0	80	3232	1910	1322	671	1239	0.351309	723	42	0.964	0.0001	0.035031	n.d.	66.9098	0.076426	145.9727	0.6171	0.6795	0	0.6795	-1.8808	*	0.1349	n.s.	-0.8625	n.s.	5.966
muxC	Pseudomo	1-3222	80	0	80	3222	2982	240	947	2035	0.317572	979	52	0.981	0.00004	0.013002	n.d.	38.7709	0.066284	197.6588	0.7619	0.6796	0	0.6796	-2.7951	***	-8.7347	**	-7.4621	**	-3.471
muxB	Pseudomo	1-3219	80	0	80	3219	2853	366	867	1986	0.303891	894	45	0.974	0.00005	0.00993	n.d.	28.3301	0.063266	180.4974	0.8634	0.6705	0	0.6705	-2.9303	***	-9.1813	**	-7.8412	**	-2.607
mexN	Pseudomo	1-3187	80	0	80	3187	3050	137	1079	1971	0.35377	1130	45	0.977	0.00004	0.036639	n.d.	111.7481	0.074802	228.1455	0.7684	0.6772	0	0.6772	-1.7749	#	1.2992	n.s.	-0.0064	n.s.	10.904
mexI	Pseudomo	1-3169	80	0	80	3169	2978	191	1003	1975	0.336803	1044	44	0.975	0.00005	0.011541	n.d.	34.3684	0.07078	210.7822	0.8382	0.6788	0	0.6788	-2.9109	***	-9.0821	**	-7.7602	**	-0.711
mexF	Pseudomo	1-3271	80	0	80	3271	1865	1406	741	1124	0.397319	854	38	0.963	0.00009	0.016622	n.d.	31.0003	0.092451	172.4215	0.4994	0.6604	0	0.6604	-2.8504	***	-6.212	**	-5.7473	**	0.94
mexD	Pseudomo	1-3132	80	0	80	3132	2190	942	235	1955	0.107306	241	47	0.978	0.00004	0.007455	n.d.	16.3269	0.022218	48.6576	0.4034	0.6516	0	0.6516	-2.2842	**	-5.7452	**	-5.1604	**	-11.383
mexW	Pseudomo	1-3057	80	0	80	3057	3057	0	210	2847	0.068695	221	44	0.976	0.00004	0.006073	n.d.	18.5652	0.014596	44.6196	0.4064	0.6452	0	0.6452	-2.0046	*	-5.7539	**	-5.0294	**	-6.557
triC	Pseudomo	1-3072	80	0	80	3072	3048	24	171	2877	0.056102	175	46	0.976	0.00005	0.005733	n.d.	17.4744	0.011592	35.3323	0.2645	0.6572	0	0.6572	-1.7278	#	-5.0974	**	-4.4399	**	-9.202
pmpM	Pseudomo	1-1434	80	0	80	1434	408	1026	30	378	0.073529	30	18	0.906	0.00017	0.007292	n.d.	2.9753	0.014845	6.057	0.0844	0.704	0	0.704	-1.5896	#	-4.3231	**	-3.9277	**	-5.089
tetA	Pseudomo	1-1571	80	0	80	1571	324	1247	208	116	0.641975	270	18	0.817	0.00115	0.196902	n.d.	63.7962	0.168249	54.5126	0.3214	0.6904	0	0.6904	0.5864	n.s.	-3.5851	**	-2.2069	#	39.764
oprN	Pseudomo	1-1529	80	0	80	1529	1293	236	488	805	0.377417	515	38	0.951	0.00016	0.022715	n.d.	29.3706	0.080416	103.9778	0.7813	0.721	0	0.721	-2.4865	**	1.4499	#	-0.2545	n.s.	0.572

opmD	Pseudomo	1-1538	80	0	80	1538	1345	193	548	797	0.407435	594	34	0.947	0.00015	0.015666	n.d.	21.0706	0.089166	119.9278	0.6786	0.7264	0	0.7264	-2.8593	***	-8.7116	**	-7.4883	**	-0.038
opmE	Pseudomo	1-1720	80	0	80	1720	1352	368	649	703	0.48003	738	41	0.97	0.00007	0.029493	n.d.	39.8741	0.110208	149.0012	0.5811	0.7121	0	0.7121	-2.5435	***	-0.5934	n.s.	-1.7036	n.s.	1.489
oprA	Pseudomo	1-1517	80	0	80	1517	1323	194	688	635	0.52003	813	36	0.956	0.00011	0.023844	n.d.	31.5462	0.124069	164.1436	0.4865	0.6899	0	0.6899	-2.8067	***	-7.2639	**	-6.4534	**	1.924
oprJ	Pseudomo	1-1495	80	0	80	1495	1400	95	592	808	0.422857	628	39	0.969	0.00005	0.017406	n.d.	24.3677	0.090566	126.7924	0.6395	0.6968	0	0.6968	-2.803	***	-8.151	**	-7.0701	**	-1.068
opmH	Pseudomo	1-1493	80	0	80	1493	1431	62	90	1341	0.062893	101	30	0.933	0.00024	0.016257	n.d.	23.2639	0.01425	20.3918	0.2129	0.6802	0	0.6802	0.4744	n.s.	-2.3988	*	-1.4721	n.s.	2.382
czcC	Pseudomo	1-1293	80	0	80	1293	1287	6	127	1160	0.098679	133	42	0.973	0.00005	0.007114	n.d.	9.1557	0.020864	26.8525	0.4023	0.7148	0	0.7148	-2.2385	**	-6.017	**	-5.3537	**	-16.667
opmB	Pseudomo	1-1500	80	0	80	1500	1497	3	82	1415	0.054776	83	43	0.972	0.00006	0.009218	n.d.	13.7997	0.011194	16.7576	0.1661	0.6958	0	0.6958	-0.5902	n.s.	-3.3505	**	-2.6795	*	-10.105
oprM	Pseudomo	1-1458	80	0	80	1458	1457	1	75	1382	0.051476	76	36	0.956	0.00011	0.007236	n.d.	10.5424	0.010531	15.3443	0.1777	0.6851	0	0.6851	-1.0424	n.s.	-3.9722	**	-3.3483	**	-7.485
mexH	Pseudomo	1-3201	80	0	80	3201	3034	167	1084	1950	0.357284	1155	52	0.98	0.00005	0.028957	n.d.	87.8554	0.07686	233.193	0.726	0.683	0	0.683	-2.1684	**	0.9793	n.s.	-0.4281	n.s.	2.52
mexX	Pseudomo	1-1256	80	0	80	1256	1005	251	383	622	0.381095	407	38	0.967	0.00006	0.022181	n.d.	22.2918	0.081764	82.1728	0.6923	0.7255	0	0.7255	-2.5205	***	0.3391	n.s.	-1.0387	n.s.	-1.276
mexP	Pseudomo	1-1274	80	0	80	1274	1003	271	426	577	0.424726	460	34	0.947	0.00014	0.018586	n.d.	18.6415	0.092596	92.8734	0.6332	0.6933	0	0.6933	-2.7675	***	-7.8918	**	-6.8797	**	-0.836
mexA	Pseudomo	1-1391	80	0	80	1391	1014	377	389	625	0.383629	407	32	0.938	0.00021	0.055052	n.d.	55.8231	0.081038	82.1728	0.8319	0.6765	0	0.6765	-1.1091	n.s.	1.8814	**	0.7466	n.s.	12.495
mexC	Pseudomo	1-1225	80	0	80	1225	1001	224	402	599	0.401598	443	31	0.946	0.00014	0.017227	n.d.	17.2443	0.089352	89.4411	0.6574	0.7143	0	0.7143	-2.794	***	-8.0048	**	-6.9726	**	-0.121
mexM	Pseudomo	1-1305	80	0	80	1305	1001	304	434	567	0.433566	468	25	0.919	0.00033	0.043719	n.d.	43.7627	0.094394	94.4886	0.6903	0.6902	0	0.6902	-1.8591	*	0.4933	n.s.	-0.5989	n.s.	16.203
triB	Pseudomo	1-1071	80	0	80	1071	1071	0	90	981	0.084034	90	34	0.962	0.00006	0.006632	n.d.	7.1028	0.016966	18.1709	0.312	0.7083	0	0.7083	-2.0432	*	-6.179	**	-5.3984	**	-11.325
triA	Pseudomo	1-1173	80	0	80	1173	1104	69	88	1016	0.07971	90	28	0.932	0.0002	0.005972	n.d.	6.5927	0.016459	18.1709	0.4614	0.7079	0	0.7079	-2.1374	*	-6.4281	**	-5.6218	**	-6.273
mexV	Pseudomo	1-1131	80	0	80	1131	1116	15	101	1015	0.090502	105	29	0.951	0.00009	0.007619	n.d.	8.5032	0.018996	21.1994	0.4544	0.6676	0	0.6676	-2.02	*	-5.5906	**	-4.958	**	-4.403
mexJ	Pseudomo	1-1104	80	0	80	1104	1104	0	55	1049	0.049819	57	31	0.916	0.00045	0.006896	n.d.	7.6136	0.010424	11.5082	0.0984	0.7095	0	0.7095	-1.1109	n.s.	-3.5854	**	-3.1205	**	-7.232
czcB	Pseudomo	1-1455	80	0	80	1455	1311	144	87	1224	0.066362	90	27	0.921	0.00033	0.004078	n.d.	5.3468	0.01386	18.1709	0.3605	0.7002	0	0.7002	-2.3674	**	-5.4315	**	-5.0236	**	-7.797
muxA	Pseudomo	1-1281	80	0	80	1281	1191	90	64	1127	0.053736	65	39	0.97	0.00005	0.004821	n.d.	5.7424	0.011019	13.1234	0.1678	0.6826	0	0.6826	-1.8595	*	-4.4252	**	-4.076	**	-22.517
mexE	Pseudomo	1-1245	80	0	80	1245	1146	99	50	1096	0.04363	51	30	0.9	0.00068	0.004528	n.d.	5.1892	0.008985	10.2968	0.1552	0.6945	0	0.6945	-1.6174	#	-3.3726	**	-3.212	**	-11.542
emrE	Pseudomo	1-333	80	0	80	333	327	6	28	299	0.085627	30	16	0.804	0.00106	0.009564	n.d.	3.1275	0.018523	6.057	0.6587	0.662	0	0.662	-1.5111	n.s.	-5.2584	**	-4.5857	**	-3.072
mexG	Pseudomo	1-1256	80	0	80	1256	1005	251	383	622	0.381095	407	38	0.967	0.00006	0.022181	n.d.	22.2918	0.081764	82.1728	0.6923	0.7255	0	0.7255	-2.5205	***	0.3391	n.s.	-1.0387	n.s.	-1.276
sav1866	Staphylocc	1-1831	158	0	158	1831	1108	723	441	667	0.398014	460	30	0.811	0.00074	0.010341	n.d.	11.4577	0.073654	81.6089	0.8196	0.3432	0	0.3432	-2.8037	***	-12.1817	**	-9.1882	**	0.634
mepA	Staphylocc	1-1356	158	0	158	1356	1356	0	96	1260	0.070796	103	31	0.811	0.0008	0.008881	n.d.	12.0422	0.013476	18.2733	0.0693	0.3292	0	0.3292	-1.0777	n.s.	-3.3609	**	-2.7899	*	0.631
lmrS	Staphylocc	1-1520	158	0	158	1520	407	1113	200	207	0.4914	231	19	0.868	0.00015	0.061306	n.d.	24.9517	0.100692	40.9818	0.5471	0.3184	0	0.3184	-1.2641	n.s.	2.3797	**	0.7975	n.s.	24.331
norA	Staphylocc	1-1522	158	0	158	1522	1088	434	497	591	0.456801	545	28	0.79	0.0006	0.033141	n.d.	36.0571	0.088868	96.6888	0.5937	0.3337	0	0.3337	-2.0483	*	-9.2032	**	-6.8881	**	23.36
norC	Staphylocc	1-1404	158	0	158	1404	1197	207	396	801	0.330827	445	31	0.862	0.0003	0.022769	n.d.	27.2546	0.065955	78.9477	0.6945	0.3659	0	0.3659	-2.1349	*	0.8805	n.s.	-0.6687	n.s.	11.823
norB	Staphylocc	1-1539	158	0	158	1539	1374	165	516	858	0.375546	614	38	0.832	0.00058	0.017753	n.d.	24.392	0.07928	108.9301	0.4612	0.3286	0	0.3286	-2.5372	***	-7.368	**	-6.0219	**	4.603
sdrM	Staphylocc	1-1347	158	0	158	1347	447	900	104	343	0.232662	114	22	0.778	0.00073	0.052112	n.d.	23.294	0.045246	20.2248	0.5484	0.305	0	0.305	0.4814	n.s.	0.5682	n.s.	0.6337	n.s.	17.913
mdeA	Staphylocc	1-1462	158	0	158	1462	1358	104	137	1221	0.100884	147	31	0.851	0.0004	0.010367	n.d.	14.0787	0.019204	26.0794	0.1223	0.3439	0	0.3439	-1.4718	n.s.	-1.2287	n.s.	-1.6059	n.s.	1.868
tet38	Staphylocc	1-1359	158	0	158	1359	1353	6	72	1281	0.053215	76	24	0.827	0.00048	0.008003	n.d.	10.8279	0.009965	13.4832	0.1263	0.3197	0	0.3197	-0.6139	n.s.	-3.9307	**	-2.9511	*	2.74
