# Supplementary material for: Quantifying the Evolutionary Conservation of Genes Encoding Multidrug Efflux Pumps in the ESKAPE Pathogens To Identify Antimicrobial Drug Targets
Source: mSystems. 2018 Apr 17;3(3):e00024-18. doi: 10.1128/mSystems.00024-18 (PMC5904435; doi:10.1128/mSystems.00024-18)
Supplement: TABLE S3 [file sys003182224st3.pdf]

Table S3: Normalized Robinson-Foulds scores for pairwise comparisons between phylogenies for each gene in each species.

Enterococcus faecium

	efrA	efrB	efmA	msrC
efrA	0	0.85	1	1
efrB	0.85	0	1	1
efmA	1	1	0	1
msrC	1	1	1	0

Staphylococcus aureus

	lmrS	norA	norB	norC	sdrM	mdeA	mepA	sav1866	tet38
lmrS	0	0.967742	0.935484	0.941935	0.923	0.935	0.923	0.948	0.941935
norA	0.967742	0	0.948387	0.980645	0.961	0.942	0.948	0.955	0.974194
norB	0.935484	0.948387	0	0.929032	0.942	0.929	0.923	0.935	0.929032
norC	0.941935	0.980645	0.929032	0	0.961	0.916	0.942	0.968	0.935484
sdrM	0.922581	0.96129	0.941935	0.96129	0.000	0.916	0.948	0.942	0.941935
mdeA	0.935484	0.941935	0.929032	0.916129	0.916	0.000	0.942	0.961	0.954839
mepA	0.922581	0.948387	0.922581	0.941935	0.948	0.942	0.000	0.948	0.941935
sav1866	0.948387	0.954839	0.935484	0.967742	0.942	0.961	0.948	0.000	0.96129
tet38	0.941935	0.974194	0.929032	0.935484	0.942	0.955	0.942	0.961	0

Klebsiella pneumoniae

	acrA	acrB	tolC	eefA	eefB	eefC	acrE	acrF	acrD	oqxA	oqxB	mdtA	mdtB	mdtC	cusA	cusB	cusF	cusC
acrA	0	0.960526	0.934211	0	0.960526	0.960526	0	0.960526	0.960526	0.947368	0.973684	0.947368	0.934211	0.934211	0.960526	0.973684	0.986842	0.960526
acrB	0.960526	0	0.934211	0.960526	0	0.960526	0.960526	0	0	0.907895	0.934211	0.907895	0.934211	0.934211	0.960526	0.973684	0.973684	0.960526
tolC	0.934211	0.934211	0	0.934211	0.934211	0.947368	0.934211	0.934211	0.934211	0.947368	0.947368	0.947368	0.921053	0.921053	0.894737	0.947368	0.986842	0.947368
eefA	0	0.960526	0.934211	0	0.960526	0.960526	0	0.960526	0.960526	0.947368	0.973684	0.947368	0.934211	0.934211	0.960526	0.973684	0.986842	0.960526
eefB	0.960526	0	0.934211	0.960526	0	0.960526	0.960526	0	0	0.907895	0.934211	0.907895	0.934211	0.934211	0.960526	0.973684	0.973684	0.960526
eefC	0.960526	0.960526	0.947368	0.960526	0.960526	0	0.960526	0.960526	0.960526	0.907895	0.894737	0.907895	0.934211	0.934211	0.934211	0.921053	1	0
acrE	0	0.960526	0.934211	0	0.960526	0.960526	0	0.960526	0.960526	0.947368	0.973684	0.947368	0.934211	0.934211	0.960526	0.973684	0.986842	0.960526
acrF	0.960526	0	0.934211	0.960526	0	0.960526	0.960526	0	0	0.907895	0.934211	0.907895	0.934211	0.934211	0.960526	0.973684	0.973684	0.960526
acrD	0.960526	0	0.934211	0.960526	0	0.960526	0.960526	0	0	0.907895	0.934211	0.907895	0.934211	0.934211	0.960526	0.973684	0.973684	0.960526
oqxA	0.947368	0.907895	0.947368	0.947368	0.907895	0.907895	0.947368	0.907895	0.907895	0	0.894737	0	0.921053	0.921053	0.947368	0.934211	0.986842	0.907895
oqxB	0.973684	0.934211	0.947368	0.973684	0.934211	0.894737	0.973684	0.934211	0.934211	0.894737	0	0.894737	0.921053	0.921053	0.960526	0.947368	1	0.894737
mdtA	0.947368	0.907895	0.947368	0.947368	0.907895	0.907895	0.947368	0.907895	0.907895	0	0.894737	0	0.921053	0.921053	0.947368	0.934211	0.986842	0.907895
mdtB	0.934211	0.934211	0.921053	0.934211	0.934211	0.934211	0.934211	0.934211	0.934211	0.921053	0.921053	0.921053	0	0	0.960526	0.986842	0.986842	0.934211
mdtC	0.934211	0.934211	0.921053	0.934211	0.934211	0.934211	0.934211	0.934211	0.934211	0.921053	0.921053	0.921053	0	0	0.960526	0.986842	0.986842	0.934211
cusA	0.960526	0.960526	0.894737	0.960526	0.960526	0.934211	0.960526	0.960526	0.960526	0.947368	0.960526	0.947368	0.960526	0.960526	0	0.868421	0.986842	0.934211
cusB	0.973684	0.973684	0.947368	0.973684	0.973684	0.921053	0.973684	0.973684	0.973684	0.934211	0.947368	0.934211	0.986842	0.986842	0.868421	0	0.986842	0.921053
cusF	0.986842	0.973684	0.986842	0.986842	0.973684	1	0.986842	0.973684	0.973684	0.986842	1	0.986842	0.986842	0.986842	0.986842	0.986842	0	1
cusC	0.960526	0.960526	0.947368	0.960526	0.960526	0	0.960526	0.960526	0.960526	0.907895	0.894737	0.907895	0.934211	0.934211	0.934211	0.921053	1	0

Acinetobacter baumannii

	adeA	adeB	adeC	adeF	adeG	adeH	adeI	adeJ	adeK	amvA	craA	cmlA	floR	tetA	abeM	abeS	qacE	smr	macA	macB	tolC	aceI
adeA	0	0.894737	0.934211	0.934211	0.947368	0.934211	0.934211	0.934211	0.934211	0.921053	0.960526	0.973684	0.907895	0.947368	0.921053	0.947368	0.960526	0.934211	0.894737	0.934211	0.921053	0.921053
adeB	0.894737	0	0.934211	0.934211	0.934211	0.934211	0.868421	0.921053	0.921053	0.907895	0.960526	0.973684	0.947368	0.907895	0.907895	0.934211	0.934211	0.934211	0.881579	0.934211	0.907895	0.921053
adeC	0.934211	0.934211	0	0.947368	0.894737	0.921053	0.960526	0.960526	0.868421	0.868421	0.973684	0.921053	0.986842	0.934211	0.921053	0.894737	0.947368	0.921053	0.907895	0.934211	0.947368	0.960526
adeF	0.934211	0.934211	0.947368	0	0.934211	0.921053	0.960526	0.921053	0.921053	0.921053	0.960526	0.973684	0.934211	0.947368	0.921053	0.934211	0.947368	0.894737	0.934211	0.894737	0.934211	0.934211
adeG	0.947368	0.934211	0.894737	0.934211	0	0.921053	0.973684	0.934211	0.894737	0.881579	0.960526	0.947368	0.973684	0.960526	0.907895	0.894737	0.934211	0.907895	0.921053	0.868421	0.947368	0.960526
adeH	0.934211	0.934211	0.921053	0.921053	0.921053	0	0.986842	0.934211	0.947368	0.907895	0.894737	0.947368	0.986842	0.973684	0.907895	0.868421	0.973684	0.934211	0.907895	0.934211	0.921053	0.960526
adeI	0.934211	0.868421	0.960526	0.960526	0.973684	0.986842	0	0.960526	0.947368	0.947368	0.960526	0.973684	0.934211	0.934211	0.921053	0.960526	0.986842	0.960526	0.921053	0.934211	0.934211	0.947368
adeJ	0.934211	0.921053	0.960526	0.921053	0.934211	0.934211	0.960526	0	0.921053	0.907895	0.921053	0.973684	0.960526	0.934211	0.934211	0.921053	0.934211	0.907895	0.921053	0.907895	0.934211	0.973684
adeK	0.934211	0.921053	0.868421	0.921053	0.894737	0.947368	0.947368	0.921053	0	0.868421	0.960526	0.960526	0.973684	0.934211	0.907895	0.907895	0.947368	0.881579	0.907895	0.868421	0.947368	0.921053
amvA	0.921053	0.907895	0.868421	0.921053	0.881579	0.907895	0.947368	0.907895	0.868421	0	0.921053	0.960526	0.947368	0.934211	0.868421	0.907895	0.934211	0.907895	0.881579	0.894737	0.921053	0.947368
craA	0.960526	0.960526	0.973684	0.960526	0.960526	0.894737	0.960526	0.921053	0.960526	0.921053	0	0.960526	0.986842	0.973684	0.947368	0.934211	0.973684	0.947368	0.934211	0.947368	0.934211	0.973684
cmlA	0.973684	0.973684	0.921053	0.973684	0.947368	0.947368	0.973684	0.973684	0.960526	0.960526	0.960526	0	0.763158	0.960526	0.973684	0.947368	0.934211	0.973684	0.960526	0.973684	0.960526	0.973684
floR	0.907895	0.947368	0.986842	0.934211	0.973684	0.986842	0.934211	0.960526	0.973684	0.947368	0.986842	0.763158	0	0.947368	0.947368	0.973684	0.934211	0.973684	0.960526	0.986842	0.986842	0.973684
tetA	0.947368	0.907895	0.934211	0.947368	0.960526	0.973684	0.934211	0.934211	0.934211	0.934211	0.973684	0.960526	0.947368	0	0.947368	0.947368	0.947368	0.960526	0.947368	0.934211	0.934211	0.947368
abeM	0.921053	0.907895	0.921053	0.921053	0.907895	0.907895	0.921053	0.934211	0.907895	0.868421	0.947368	0.973684	0.947368	0.947368	0	0.921053	0.921053	0.921053	0.894737	0.894737	0.921053	0.947368
abeS	0.947368	0.934211	0.894737	0.934211	0.894737	0.868421	0.960526	0.921053	0.907895	0.907895	0.934211	0.947368	0.973684	0.947368	0.921053	0	0.921053	0.921053	0.907895	0.868421	0.934211	0.934211
qacE	0.960526	0.934211	0.947368	0.947368	0.934211	0.973684	0.986842	0.934211	0.947368	0.934211	0.973684	0.934211	0.934211	0.947368	0.921053	0.921053	0	0.921053	0.947368	0.934211	0.947368	0.947368
smr	0.934211	0.934211	0.921053	0.894737	0.907895	0.934211	0.960526	0.907895	0.881579	0.907895	0.947368	0.973684	0.973684	0.960526	0.921053	0.921053	0.921053	0	0.921053	0.868421	0.921053	0.921053
macA	0.894737	0.881579	0.907895	0.934211	0.921053	0.907895	0.921053	0.921053	0.907895	0.881579	0.934211	0.960526	0.960526	0.947368	0.894737	0.907895	0.947368	0.921053	0	0.907895	0.921053	0.921053
macB	0.934211	0.934211	0.934211	0.894737	0.868421	0.934211	0.934211	0.907895	0.868421	0.894737	0.947368	0.973684	0.986842	0.934211	0.894737	0.868421	0.934211	0.868421	0.907895	0	0.934211	0.934211
tolC	0.921053	0.907895	0.947368	0.934211	0.947368	0.921053	0.934211	0.934211	0.947368	0.921053	0.934211	0.960526	0.986842	0.934211	0.921053	0.934211	0.947368	0.921053	0.921053	0.934211	0	0.960526
aceI	0.921053	0.921053	0.960526	0.934211	0.960526	0.960526	0.947368	0.973684	0.921053	0.947368	0.973684	0.973684	0.973684	0.947368	0.947368	0.934211	0.947368	0.921053	0.921053	0.934211	0.960526	0

Pseudomonas aeruginosa

	czcA	czcB	czcC	emrE	mexA	mexB	mexC	mexD	mexE	mexF	mexG	mexH	mexI	mexJ	mexK	mexM	mexN	mexP	mexQ	mexV	mexW
czcA	0	0.896104	0.844156	0.987013	0.922078	0.831169	0.896104	0.766234	0.896104	0.857143	0.844156	0.831169	0.87013	0.948052	0.766234	0.922078	0.805195	0.883117	0.792208	0.909091	0.844156
czcB	0.896104	0	0.87013	0.974026	0.922078	0.87013	0.948052	0.857143	0.896104	0.831169	0.896104	0.818182	0.896104	0.974026	0.883117	0.948052	0.87013	0.857143	0.883117	0.948052	0.831169
czcC	0.844156	0.87013	0	1	0.935065	0.883117	0.896104	0.87013	0.961039	0.857143	0.922078	0.857143	0.909091	0.961039	0.844156	0.935065	0.896104	0.87013	0.909091	0.922078	0.87013
emrE	0.987013	0.974026	1	0	0.961039	0.987013	1	0.987013	0.987013	0.987013	0.987013	0.961039	0.961039	1	0.987013	1	0.974026	0.974026	0.987013	0.987013	0.987013
mexA	0.922078	0.922078	0.935065	0.961039	0	0.87013	0.974026	0.883117	0.909091	0.857143	0.896104	0.87013	0.883117	0.961039	0.857143	0.922078	0.87013	0.948052	0.883117	0.909091	0.87013
mexB	0.831169	0.87013	0.883117	0.987013	0.87013	0	0.922078	0.792208	0.896104	0.844156	0.896104	0.844156	0.818182	0.948052	0.792208	0.87013	0.792208	0.87013	0.818182	0.896104	0.805195
mexC	0.896104	0.948052	0.896104	1	0.974026	0.922078	0	0.844156	0.935065	0.896104	0.922078	0.922078	0.922078	0.974026	0.896104	0.935065	0.935065	0.922078	0.909091	0.948052	0.896104
mexD	0.766234	0.857143	0.87013	0.987013	0.883117	0.792208	0.844156	0	0.831169	0.792208	0.844156	0.818182	0.831169	0.935065	0.779221	0.87013	0.779221	0.857143	0.818182	0.883117	0.766234
mexE	0.896104	0.896104	0.961039	0.987013	0.909091	0.896104	0.935065	0.831169	0	0.909091	0.857143	0.857143	0.909091	0.935065	0.87013	0.922078	0.87013	0.857143	0.883117	0.961039	0.831169
mexF	0.857143	0.831169	0.857143	0.987013	0.857143	0.844156	0.896104	0.792208	0.909091	0	0.909091	0.87013	0.844156	0.961039	0.818182	0.909091	0.818182	0.87013	0.844156	0.896104	0.779221
mexG	0.844156	0.896104	0.922078	0.987013	0.896104	0.896104	0.922078	0.844156	0.857143	0.909091	0	0.792208	0.896104	0.948052	0.792208	0.896104	0.896104	0.883117	0.883117	0.909091	0.857143
mexH	0.831169	0.818182	0.857143	0.961039	0.87013	0.844156	0.922078	0.818182	0.857143	0.87013	0.792208	0	0.87013	0.935065	0.805195	0.883117	0.857143	0.87013	0.883117	0.922078	0.857143
mexI	0.87013	0.896104	0.909091	0.961039	0.883117	0.818182	0.922078	0.831169	0.909091	0.844156	0.896104	0.87013	0	0.935065	0.792208	0.909091	0.831169	0.857143	0.831169	0.896104	0.831169
mexJ	0.948052	0.974026	0.961039	1	0.961039	0.948052	0.974026	0.935065	0.935065	0.961039	0.948052	0.935065	0.935065	0	0.909091	0.948052	0.948052	0.935065	0.961039	0.987013	0.922078
mexK	0.766234	0.883117	0.844156	0.987013	0.857143	0.792208	0.896104	0.779221	0.87013	0.818182	0.792208	0.805195	0.792208	0.909091	0	0.857143	0.805195	0.844156	0.779221	0.87013	0.779221
mexM	0.922078	0.948052	0.935065	1	0.922078	0.87013	0.935065	0.87013	0.922078	0.909091	0.896104	0.883117	0.909091	0.948052	0.857143	0	0.844156	0.922078	0.909091	0.961039	0.857143
mexN	0.805195	0.87013	0.896104	0.974026	0.87013	0.792208	0.935065	0.779221	0.87013	0.818182	0.896104	0.857143	0.831169	0.948052	0.805195	0.844156	0	0.857143	0.753247	0.896104	0.792208
mexP	0.883117	0.857143	0.87013	0.974026	0.948052	0.87013	0.922078	0.857143	0.857143	0.87013	0.883117	0.87013	0.857143	0.935065	0.844156	0.922078	0.857143	0	0.883117	0.935065	0.831169
mexQ	0.792208	0.883117	0.909091	0.987013	0.883117	0.818182	0.909091	0.818182	0.883117	0.844156	0.883117	0.883117	0.831169	0.961039	0.779221	0.909091	0.753247	0.883117	0	0.896104	0.818182
mexV	0.909091	0.948052	0.922078	0.987013	0.909091	0.896104	0.948052	0.883117	0.961039	0.896104	0.909091	0.922078	0.896104	0.987013	0.87013	0.961039	0.896104	0.935065	0.896104	0	0.896104
mexW	0.844156	0.831169	0.87013	0.987013	0.87013	0.805195	0.896104	0.766234	0.831169	0.779221	0.857143	0.857143	0.831169	0.922078	0.779221	0.857143	0.792208	0.831169	0.818182	0.896104	0
mexX	0.844156	0.896104	0.922078	0.987013	0.896104	0.896104	0.922078	0.844156	0.857143	0.909091	0	0.792208	0.896104	0.948052	0.792208	0.896104	0.896104	0.883117	0.883117	0.909091	0.857143
mexY	0.831169	0.818182	0.857143	0.961039	0.87013	0.844156	0.922078	0.818182	0.857143	0.87013	0.792208	0	0.87013	0.935065	0.805195	0.883117	0.857143	0.87013	0.883117	0.922078	0.857143
muxA	0.818182	0.909091	0.87013	0.948052	0.922078	0.805195	0.909091	0.857143	0.922078	0.844156	0.896104	0.857143	0.896104	0.961039	0.857143	0.896104	0.87013	0.909091	0.896104	0.935065	0.87013
muxB	0.831169	0.909091	0.896104	0.987013	0.857143	0.87013	0.922078	0.792208	0.883117	0.87013	0.896104	0.831169	0.896104	0.948052	0.844156	0.922078	0.87013	0.909091	0.844156	0.896104	0.844156
muxC	0.818182	0.831169	0.844156	0.987013	0.857143	0.844156	0.909091	0.818182	0.87013	0.818182	0.844156	0.844156	0.87013	0.961039	0.818182	0.909091	0.818182	0.883117	0.805195	0.883117	0.805195
opmB	0.87013	0.883117	0.896104	0.987013	0.909091	0.857143	0.896104	0.805195	0.935065	0.87013	0.909091	0.883117	0.922078	0.987013	0.831169	0.883117	0.857143	0.948052	0.896104	0.922078	0.87013
opmD	0.922078	0.935065	0.922078	0.987013	0.896104	0.805195	0.974026	0.87013	0.909091	0.896104	0.909091	0.896104	0.818182	0.948052	0.857143	0.909091	0.87013	0.896104	0.883117	0.948052	0.87013
opmE	0.883117	0.883117	0.935065	0.987013	0.883117	0.805195	0.948052	0.818182	0.87013	0.896104	0.87013	0.844156	0.883117	0.948052	0.818182	0.896104	0.844156	0.883117	0.844156	0.909091	0.883117
opmH	0.896104	0.935065	0.922078	0.974026	0.909091	0.87013	0.935065	0.883117	0.935065	0.935065	0.909091	0.909091	0.844156	0.948052	0.883117	0.935065	0.909091	0.909091	0.922078	0.909091	0.87013
oprA	0.883117	0.857143	0.948052	0.961039	0.896104	0.844156	0.961039	0.844156	0.831169	0.922078	0.87013	0.831169	0.883117	0.948052	0.857143	0.896104	0.883117	0.883117	0.909091	0.935065	0.883117
oprJ	0.857143	0.896104	0.857143	1	0.935065	0.883117	0.922078	0.857143	0.896104	0.844156	0.909091	0.883117	0.883117	0.948052	0.805195	0.909091	0.857143	0.87013	0.87013	0.948052	0.87013
oprM	0.883117	0.909091	0.922078	0.961039	0.857143	0.857143	0.935065	0.857143	0.857143	0.909091	0.87013	0.831169	0.87013	0.935065	0.818182	0.883117	0.87013	0.87013	0.896104	0.935065	0.857143
oprN	0.831169	0.896104	0.909091	0.974026	0.935065	0.922078	0.935065	0.857143	0.948052	0.844156	0.948052	0.922078	0.909091	0.948052	0.844156	0.961039	0.87013	0.883117	0.857143	0.935065	0.87013

	mexX	mexY	muxA	muxB	muxC	opmB	opmD	opmE	opmH	oprA	oprJ	oprM	oprN	pmpM	tetA	triA	triB	triC
czcA	0.844156	0.831169	0.818182	0.831169	0.818182	0.87013	0.922078	0.883117	0.896104	0.883117	0.857143	0.883117	0.831169	0.883117	0.896104	0.883117	0.935065	0.844156
czcB	0.896104	0.818182	0.909091	0.909091	0.831169	0.883117	0.935065	0.883117	0.935065	0.857143	0.896104	0.909091	0.896104	0.857143	0.935065	0.948052	0.935065	0.857143
czcC	0.922078	0.857143	0.87013	0.896104	0.844156	0.896104	0.922078	0.935065	0.922078	0.948052	0.857143	0.922078	0.909091	0.896104	0.935065	0.922078	0.896104	0.857143
emrE	0.987013	0.961039	0.948052	0.987013	0.987013	0.987013	0.987013	0.987013	0.974026	0.961039	1	0.961039	0.974026	0.974026	0.987013	0.987013	1	0.961039
mexA	0.896104	0.87013	0.922078	0.857143	0.857143	0.909091	0.896104	0.883117	0.909091	0.896104	0.935065	0.857143	0.935065	0.909091	0.909091	0.935065	0.974026	0.883117
mexB	0.896104	0.844156	0.805195	0.87013	0.844156	0.857143	0.805195	0.805195	0.87013	0.844156	0.883117	0.857143	0.922078	0.857143	0.909091	0.922078	0.948052	0.844156
mexC	0.922078	0.922078	0.909091	0.922078	0.909091	0.896104	0.974026	0.948052	0.935065	0.961039	0.922078	0.935065	0.935065	0.948052	0.948052	0.948052	0.948052	0.896104
mexD	0.844156	0.818182	0.857143	0.792208	0.818182	0.805195	0.87013	0.818182	0.883117	0.844156	0.857143	0.857143	0.857143	0.883117	0.896104	0.922078	0.922078	0.831169
mexE	0.857143	0.857143	0.922078	0.883117	0.87013	0.935065	0.909091	0.87013	0.935065	0.831169	0.896104	0.857143	0.948052	0.922078	0.935065	0.935065	0.935065	0.922078
mexF	0.909091	0.87013	0.844156	0.87013	0.818182	0.87013	0.896104	0.896104	0.935065	0.922078	0.844156	0.909091	0.844156	0.857143	0.883117	0.935065	0.922078	0.831169
mexG	0	0.792208	0.896104	0.896104	0.844156	0.909091	0.909091	0.87013	0.909091	0.87013	0.909091	0.87013	0.948052	0.935065	0.896104	0.961039	0.922078	0.883117
mexH	0.792208	0	0.857143	0.831169	0.844156	0.883117	0.896104	0.844156	0.909091	0.831169	0.883117	0.831169	0.922078	0.857143	0.909091	0.909091	0.935065	0.857143
mexI	0.896104	0.87013	0.896104	0.896104	0.87013	0.922078	0.818182	0.883117	0.844156	0.883117	0.883117	0.87013	0.909091	0.883117	0.922078	0.896104	0.922078	0.792208
mexJ	0.948052	0.935065	0.961039	0.948052	0.961039	0.987013	0.948052	0.948052	0.948052	0.948052	0.948052	0.935065	0.948052	0.935065	0.974026	0.974026	0.974026	0.948052
mexK	0.792208	0.805195	0.857143	0.844156	0.818182	0.831169	0.857143	0.818182	0.883117	0.857143	0.805195	0.818182	0.844156	0.844156	0.883117	0.922078	0.883117	0.818182
mexM	0.896104	0.883117	0.896104	0.922078	0.909091	0.883117	0.909091	0.896104	0.935065	0.896104	0.909091	0.883117	0.961039	0.896104	0.935065	0.961039	0.948052	0.896104
mexN	0.896104	0.857143	0.87013	0.87013	0.818182	0.857143	0.87013	0.844156	0.909091	0.883117	0.857143	0.87013	0.87013	0.857143	0.935065	0.935065	0.922078	0.831169
mexP	0.883117	0.87013	0.909091	0.909091	0.883117	0.948052	0.896104	0.883117	0.909091	0.883117	0.87013	0.87013	0.883117	0.896104	0.961039	0.935065	0.896104	0.909091
mexQ	0.883117	0.883117	0.896104	0.844156	0.805195	0.896104	0.883117	0.844156	0.922078	0.909091	0.87013	0.896104	0.857143	0.883117	0.935065	0.935065	0.922078	0.831169
mexV	0.909091	0.922078	0.935065	0.896104	0.883117	0.922078	0.948052	0.909091	0.909091	0.935065	0.948052	0.935065	0.935065	0.948052	0.948052	0.948052	0.935065	0.896104
mexW	0.857143	0.857143	0.87013	0.844156	0.805195	0.87013	0.87013	0.883117	0.87013	0.883117	0.87013	0.857143	0.87013	0.857143	0.909091	0.922078	0.922078	0.831169
mexX	0	0.792208	0.896104	0.896104	0.844156	0.909091	0.909091	0.87013	0.909091	0.87013	0.909091	0.87013	0.948052	0.935065	0.896104	0.961039	0.922078	0.883117
mexY	0.792208	0	0.857143	0.831169	0.844156	0.883117	0.896104	0.844156	0.909091	0.831169	0.883117	0.831169	0.922078	0.857143	0.909091	0.909091	0.935065	0.857143
muxA	0.896104	0.857143	0	0.896104	0.857143	0.87013	0.909091	0.909091	0.909091	0.909091	0.883117	0.87013	0.948052	0.896104	0.909091	0.935065	0.922078	0.844156
muxB	0.896104	0.831169	0.896104	0	0.818182	0.883117	0.87013	0.87013	0.922078	0.909091	0.896104	0.922078	0.896104	0.909091	0.883117	0.909091	0.948052	0.883117
muxC	0.844156	0.844156	0.857143	0.818182	0	0.87013	0.935065	0.857143	0.87013	0.87013	0.844156	0.87013	0.883117	0.87013	0.883117	0.922078	0.935065	0.818182
opmB	0.909091	0.883117	0.87013	0.883117	0.87013	0	0.922078	0.857143	0.974026	0.922078	0.922078	0.922078	0.909091	0.922078	0.909091	0.948052	0.961039	0.857143
opmD	0.909091	0.896104	0.909091	0.87013	0.935065	0.922078	0	0.883117	0.896104	0.896104	0.909091	0.909091	0.935065	0.935065	0.948052	0.935065	0.909091	0.883117
opmE	0.87013	0.844156	0.909091	0.87013	0.857143	0.857143	0.883117	0	0.935065	0.844156	0.883117	0.87013	0.935065	0.909091	0.922078	0.948052	0.961039	0.896104
opmH	0.909091	0.909091	0.909091	0.922078	0.87013	0.974026	0.896104	0.935065	0	0.935065	0.935065	0.909091	0.974026	0.935065	0.948052	0.922078	0.961039	0.883117
oprA	0.87013	0.831169	0.909091	0.909091	0.87013	0.922078	0.896104	0.844156	0.935065	0	0.909091	0.493506	0.948052	0.87013	0.935065	0.948052	0.961039	0.922078
oprJ	0.909091	0.883117	0.883117	0.896104	0.844156	0.922078	0.909091	0.883117	0.935065	0.909091	0	0.909091	0.896104	0.87013	0.961039	0.948052	0.896104	0.883117
oprM	0.87013	0.831169	0.87013	0.922078	0.87013	0.922078	0.909091	0.87013	0.909091	0.493506	0.909091	0	0.948052	0.883117	0.909091	0.909091	0.922078	0.909091
oprN	0.948052	0.922078	0.948052	0.896104	0.883117	0.909091	0.935065	0.935065	0.974026	0.948052	0.896104	0.948052	0	0.896104	0.961039	0.974026	0.948052	0.896104

pmpM	0.883117	0.857143	0.896104	0.974026	0.909091	0.857143	0.948052	0.883117	0.922078	0.857143	0.935065	0.857143	0.883117	0.935065	0.844156	0.896104	0.857143	0.896104	0.883117	0.948052	0.857143	0.935065	0.857143	0.896104	0.909091	0.87013	0.922078	0.935065	0.909091	0.935065	0.87013	0.87013	0.883117	0.896104	0	0.948052	0.974026	0.974026	0.857143
tetA	0.896104	0.935065	0.935065	0.987013	0.909091	0.909091	0.948052	0.896104	0.935065	0.883117	0.896104	0.909091	0.922078	0.974026	0.883117	0.935065	0.935065	0.961039	0.935065	0.948052	0.909091	0.896104	0.909091	0.909091	0.883117	0.883117	0.909091	0.948052	0.922078	0.948052	0.935065	0.961039	0.909091	0.961039	0.948052	0	0.922078	0.948052	0.909091
triA	0.883117	0.948052	0.922078	0.987013	0.935065	0.922078	0.948052	0.922078	0.935065	0.935065	0.961039	0.909091	0.896104	0.974026	0.922078	0.961039	0.935065	0.935065	0.935065	0.948052	0.922078	0.961039	0.909091	0.935065	0.909091	0.922078	0.948052	0.935065	0.948052	0.922078	0.948052	0.948052	0.909091	0.974026	0.974026	0.922078	0	0.922078	0.935065
triB	0.935065	0.935065	0.896104	1	0.974026	0.948052	0.948052	0.922078	0.935065	0.922078	0.922078	0.935065	0.922078	0.974026	0.883117	0.948052	0.922078	0.896104	0.922078	0.935065	0.922078	0.922078	0.935065	0.922078	0.948052	0.935065	0.961039	0.909091	0.961039	0.961039	0.961039	0.896104	0.922078	0.948052	0.974026	0.948052	0.922078	0	0.87013
triC	0.844156	0.857143	0.857143	0.961039	0.883117	0.844156	0.896104	0.831169	0.922078	0.831169	0.883117	0.857143	0.792208	0.948052	0.818182	0.896104	0.831169	0.909091	0.831169	0.896104	0.831169	0.883117	0.857143	0.844156	0.883117	0.818182	0.857143	0.883117	0.896104	0.883117	0.922078	0.883117	0.909091	0.896104	0.857143	0.909091	0.935065	0.87013	0

Enterobacter spp.

	acrA	acrB	tolC	eefA	eefB	eefC	acrE	acrF	acrD	oqxA	oqxB	mdtA	mdtB	mdtC	cusA	cusB	cusF	cusC
acrA	0	0.56	0.52	0.72	0.7	0.72	0.62	0.44	0.58	0.7	0.66	0.54	0.52	0.68	0.76	0.88	0.92	0.88
acrB	0.56	0	0.58	0.7	0.62	0.68	0.6	0.5	0.58	0.64	0.62	0.62	0.46	0.58	0.74	0.84	0.88	0.86
tolC	0.52	0.58	0	0.72	0.7	0.72	0.6	0.5	0.58	0.68	0.62	0.62	0.62	0.64	0.72	0.84	0.9	0.86
eefA	0.72	0.7	0.72	0	0.52	0.56	0.64	0.66	0.72	0.7	0.7	0.74	0.68	0.76	0.82	0.9	0.88	0.88
eefB	0.7	0.62	0.7	0.52	0	0.5	0.76	0.7	0.66	0.66	0.7	0.66	0.72	0.74	0.78	0.88	0.9	0.88
eefC	0.72	0.68	0.72	0.56	0.5	0	0.68	0.66	0.74	0.74	0.74	0.74	0.7	0.78	0.8	0.86	0.9	0.84
acrE	0.62	0.6	0.6	0.64	0.76	0.68	0	0.46	0.68	0.72	0.66	0.7	0.62	0.68	0.8	0.86	0.88	0.86
acrF	0.44	0.5	0.5	0.66	0.7	0.66	0.46	0	0.56	0.64	0.68	0.48	0.52	0.68	0.76	0.84	0.88	0.86
acrD	0.58	0.58	0.58	0.72	0.66	0.74	0.68	0.56	0	0.72	0.64	0.54	0.56	0.6	0.76	0.88	0.94	0.9
oqxA	0.7	0.64	0.68	0.7	0.66	0.74	0.72	0.64	0.72	0	0.4	0.62	0.64	0.64	0.78	0.9	0.92	0.88
oqxB	0.66	0.62	0.62	0.7	0.7	0.74	0.66	0.68	0.64	0.4	0	0.68	0.68	0.62	0.78	0.88	0.94	0.9
mdtA	0.54	0.62	0.62	0.74	0.66	0.74	0.7	0.48	0.54	0.62	0.68	0	0.44	0.6	0.74	0.88	0.92	0.86
mdtB	0.52	0.46	0.62	0.68	0.72	0.7	0.62	0.52	0.56	0.64	0.68	0.44	0	0.6	0.72	0.88	0.9	0.86
mdtC	0.68	0.58	0.64	0.76	0.74	0.78	0.68	0.68	0.6	0.64	0.62	0.6	0.6	0	0.74	0.84	0.92	0.86
cusA	0.76	0.74	0.72	0.82	0.78	0.8	0.8	0.76	0.76	0.78	0.78	0.74	0.72	0.74	0	0.84	0.9	0.86
cusB	0.88	0.84	0.84	0.9	0.88	0.86	0.86	0.84	0.88	0.9	0.88	0.88	0.88	0.84	0.84	0	0.56	0.66
cusF	0.92	0.88	0.9	0.88	0.9	0.9	0.88	0.88	0.94	0.92	0.94	0.92	0.9	0.92	0.9	0.56	0	0.72
cusC	0.88	0.86	0.86	0.88	0.88	0.84	0.86	0.86	0.9	0.88	0.9	0.86	0.86	0.86	0.86	0.66	0.72	0
