# Supplementary material for: Quantifying the Evolutionary Conservation of Genes Encoding Multidrug Efflux Pumps in the ESKAPE Pathogens To Identify Antimicrobial Drug Targets
Source: mSystems. 2018 Apr 17;3(3):e00024-18. doi: 10.1128/mSystems.00024-18 (PMC5904435; doi:10.1128/mSystems.00024-18)
Supplement: TABLE S4 [file sys003182224st4.pdf]

Table S4: Results from selection analyses; Stat: both p (proportion of sites which match each model of selection) and ω (d_N/d_S ratio for the variable sites which fit into each model of selection) for Purifying, Neutral and Positive, p-value – probability value for likelihood ratio test statistic.

Organism	Class	Gene	Stat	Purifying	Neutral	Positive	p-value
Enterococcus faecium	ABC	efrA	p	0.753	0.039	0.208	1.000
			ω	0.379	1.000	1.000	
		efrB	p	0.096	0.798	0.106	0.021
			ω	0.000	1.000	3.948	
	ABC	msrC	p	1.000	0.000	0.000	0.991
			ω	0.200	1.000	1.000	
	MFS	efmA	p	0.898	0.000	0.102	0.347
			ω	0.040	1.000	2.226	
Staphylococcus aureus	ABC	sav1866	p	0.588	0.000	0.412	0.215
			ω	0.338	1.000	1.522	
	MATE	mepA	p	0.957	0.038	0.005	0.010
			ω	0.043	1.000	4.825	
	MFS	lmrS	p	0.065	0.459	0.476	0.000
			ω	0.000	1.000	4.628	
		mdeA	p	0.959	0.017	0.024	1.000
			ω	0.024	1.000	1.000	
		norA	p	0.818	0.000	0.182	0.000
			ω	0.423	1.000	3.767	
		norB	p	0.940	0.040	0.020	0.000
			ω	0.039	1.000	1.000	
		norC	p	0.920	0.048	0.032	1.000
			ω	0.012	1.000	1.000	
		sdrM	p	0.948	0.046	0.006	0.995
			ω	0.051	1.000	1.000	
		tet38	p	0.973	0.024	0.003	0.184
			ω	0.022	1.000	8.087	
Klebsiella pneumoniae	Chaperone	cusF	p	0.142	0.644	0.214	1.000
			ω	0.054	1.000	1.000	
	RND- IM	acrB	p	0.994	0.000	0.006	0.220
			ω	0.000	1.000	3.304	

		acrD	p	0.994	0.000	0.006	0.220
			ω	0.000	1.000	3.304	
		acrF	p	0.994	0.000	0.006	0.220
			ω	0.000	1.000	3.304	
		cusB	p	0.762	0.147	0.091	0.125
			ω	0.391	1.000	1.633	
		eefB	p	0.994	0.000	0.006	0.220
			ω	0.000	1.000	3.304	
		mdtB	p	0.986	0.000	0.014	0.482
			ω	0.029	1.000	1.649	
		mdtC	p	0.986	0.000	0.014	0.482
			ω	0.029	1.000	1.649	
		oqxB	p	0.996	0.004	0.000	1.000
			ω	0.008	1.000	44.246	
	RND - OM	cusC	p	0.882	0.044	0.073	0.993
			ω	0.052	1.000	1.000	
		eefC	p	0.882	0.054	0.063	1.000
			ω	0.052	1.000	1.000	
		tolC	p	0.952	0.001	0.047	0.511
			ω	0.000	1.000	1.306	
	RND - PA	acrA	p	1.000	0.000	0.000	0.996
			ω	0.048	1.000	1.000	
		acrE	p	0.943	0.026	0.031	1.000
			ω	0.000	1.000	1.000	
		cusA	p	0.714	0.270	0.016	0.222
			ω	0.268	1.000	2.477	
		eefA	p	1.000	0.000	0.000	0.996
			ω	0.048	1.000	1.000	
		mdtA	p	0.990	0.007	0.003	0.057
			ω	0.021	1.000	5.569	
		oqxA	p	0.990	0.007	0.003	0.057
			ω	0.021	1.000	5.569	
Acinetobacter baumannii	ABC	macA	p	0.993	0.000	0.007	0.001
			ω	0.010	1.000	5.911	
		macB	p	0.992	0.000	0.008	1.000
			ω	0.006	1.000	1.000	
		tolC	p	0.953	0.000	0.047	0.510
			ω	0.001	1.000	1.315	
	MATE	abeM	p	1.000	0.000	0.000	0.986
			ω	0.046	1.000	1.000	
	MFS	amvA	p	0.945	0.047	0.008	1.000
			ω	0.008	1.000	1.000	
		cmlA	p	0.247	0.747	0.006	0.048

			ω	0.289	1.000	14.817	
		craA	p	0.024	0.938	0.037	0.000
			ω	0.000	1.000	31.779	
		floR	p	0.389	0.577	0.034	0.298
			ω	0.457	1.000	2.712	
		tetA	p	0.393	0.286	0.321	1.000
			ω	0.319	1.000	1.000	
	PACE	aceI	p	0.000	0.002	0.998	0.033
			ω	1.000	1.000	2.090	
	RND-IM	adeB	p	0.351	0.614	0.035	0.000
			ω	0.168	1.000	20.736	
		adeG	p	0.996	0.004	0.000	0.996
			ω	0.008	1.000	30.383	
		adeJ	p	0.994	0.000	0.006	0.220
			ω	0.000	1.000	3.304	
	RND - OM	adeC	p	0.882	0.060	0.058	0.995
			ω	0.052	1.000	1.000	
		adeH	p	0.994	0.000	0.006	1.000
			ω	0.069	1.000	1.042	
		adeK	p	0.914	0.079	0.007	0.000
			ω	0.055	1.000	10.754	
	RND - PA	adeA	p	0.390	0.544	0.066	
			ω	0.357	1.000	4.957	
		adeF	p	0.959	0.000	0.041	0.004
			ω	0.040	1.000	2.230	
		adeI	p	1.000	0.000	0.000	0.996
			ω	0.048	1.000	1.000	
	SMR	abeS	p	1.000	0.000	0.000	0.994
			ω	0.038	1.000	1.000	
		qacE	p	0.907	0.001	0.092	1.000
			ω	0.023	1.000	1.000	
		smr1	p	0.977	0.000	0.023	0.904
			ω	0.000	1.000	1.127	
Pseudomonas aeruginosa	MATE	pmpM	p	0.961	0.039	0.000	0.990
			ω	0.015	1.000	19.981	
	MFS	tetA	p	0.100	0.780	0.120	0.998
			ω	0.051	1.000	1.000	
	RND - IM	czcA	p	0.111	0.870	0.020	0.000
			ω	0.000	1.000	27.662	
		mexB	p	0.119	0.713	0.167	0.000
			ω	0.074	1.000	5.964	
		mexD	p	0.963	0.014	0.023	1.000
			ω	0.017	1.000	1.000	

	mexF	p	0.830	0.004	0.167	0.000
		ω	1.000	1.000	6.627	
	mexI	p	0.579	0.000	0.421	0.000
		ω	0.735	1.000	5.830	
	mexK	p	0.016	0.925	0.059	0.000
		ω	0.000	1.000	21.320	
	mexN	p	0.491	0.501	0.008	0.000
		ω	0.145	1.000	20.268	
	mexQ	p	0.152	0.831	0.017	0.000
		ω	0.000	1.000	32.732	
	mexW	p	0.988	0.010	0.002	0.000
		ω	0.010	1.000	29.398	
	mexY	p	0.079	0.870	0.050	0.000
		ω	0.000	1.000	14.897	
	muxB	p	0.478	0.516	0.007	0.000
		ω	0.071	1.000	39.801	
	muxC	p	0.460	0.530	0.010	0.000
		ω	0.131	1.000	20.982	
	triC	p	0.997	0.000	0.003	0.071
		ω	0.003	1.000	3.142	
RND - OM	czcC	p	0.978	0.022	0.000	1.000
		ω	0.026	1.000	1.000	
	opmB	p	0.989	0.011	0.000	1.000
		ω	0.019	1.000	31.000	
	opmD	p	0.077	0.522	0.401	0.000
		ω	0.000	1.000	4.870	
	opmE	p	0.078	0.852	0.070	0.000
		ω	0.000	1.000	12.094	
	opmH	p	0.971	0.014	0.015	1.000
		ω	0.017	1.000	1.000	
	oprA	p	0.187	0.540	0.273	0.000
		ω	0.158	1.000	6.158	
	oprJ	p	0.408	0.304	0.287	0.980
		ω	0.097	1.000	1.000	
	oprM	p	1.000	0.000	0.000	0.954
		ω	0.009	1.000	44.829	
	oprN	p	0.043	0.873	0.085	0.000
		ω	0.000	1.000	23.774	
RND - PA	czcB	p	0.975	0.010	0.014	1.000
		ω	0.009	1.000	1.000	
	mexA	p	0.282	0.218	0.500	0.000
		ω	0.907	1.000	14.201	
	mexC	p	0.438	0.537	0.025	0.000
		ω	0.082	1.000	15.131	

		mexE	p	1.000	0.000	0.000	0.966
			ω	0.021	1.000	1.000	
		mexH	p	0.079	0.870	0.050	0.000
			ω	0.000	1.000	14.897	
		mexJ	p	0.972	0.025	0.003	0.010
			ω	0.026	1.000	25.004	
		mexM	p	0.803	0.143	0.054	0.987
			ω	0.139	1.000	1.000	
		mexP	p	0.207	0.772	0.021	0.000
			ω	0.000	1.000	17.679	
		mexV	p	0.962	0.015	0.023	1.000
			ω	0.015	1.000	1.000	
		mexX	p	0.090	0.786	0.123	0.000
			ω	0.000	1.000	11.739	
		muxA	p	0.996	0.000	0.004	0.122
			ω	0.030	1.000	3.720	
		triA	p	0.960	0.028	0.012	0.002
			ω	0.025	1.000	6.211	
		triB	p	0.959	0.020	0.021	1.000
			ω	0.026	1.000	1.000	
	RND – Unknown	mexG	p	0.463	0.409	0.128	0.000
			ω	1.000	1.000	12.295	
	SMR	emrE	p	0.977	0.009	0.014	1.000
			ω	0.010	1.000	1.000	
Enterobacter spp.	Chaperone	cusF	p	0.716	0.216	0.068	0.997
			ω	0.108	1.000	1.000	
	RND - IM	acrB	p	0.746	0.244	0.010	0.000
			ω	0.067	1.000	3.436	
		acrD	p	0.962	0.033	0.005	0.992
			ω	0.021	1.000	1.000	
		acrF	p	0.202	0.488	0.310	0.000
			ω	0.056	1.000	2.700	
		cusB	p	0.469	0.265	0.266	1.000
			ω	0.200	1.000	1.000	
		eefB	p	0.119	0.483	0.399	0.000
			ω	0.042	1.000	2.914	
		mdtB	p	0.612	0.324	0.064	0.000
			ω	0.173	1.000	2.492	
		mdtC	p	0.108	0.530	0.362	0.000
			ω	0.151	1.000	3.693	
		oqxB	p	0.366	0.476	0.159	0.000
			ω	0.197	1.000	2.655	
	RND - OM	cusC	p	0.120	0.602	0.278	0.000

		ω	0.172	1.000	2.228	
	eefC	p	0.808	0.126	0.065	0.988
		ω	0.162	1.000	1.000	
	tolC	p	0.604	0.227	0.169	0.000
		ω	0.250	1.000	2.532	
RND - PA	acrA	p	0.704	0.205	0.091	1.000
		ω	0.091	1.000	1.000	
	acrE	p	0.024	0.264	0.712	0.000
		ω	0.000	1.000	3.245	
	cusA	p	0.675	0.210	0.115	1.000
		ω	0.098	1.000	1.000	
	eefA	p	0.229	0.678	0.093	0.000
		ω	0.079	1.000	2.257	
	mdtA	p	0.064	0.639	0.298	0.000
		ω	0.014	1.000	3.607	
	oqxA	p	0.560	0.425	0.015	0.001
		ω	0.201	1.000	3.823	
